# DeepCryoPicker: fully automated deep neural network for single protein particle picking in cryo-EM

**DOI:** 10.1186/s12859-020-03809-7

**Published:** 2020-11-09

**Authors:** Adil Al-Azzawi, Anes Ouadou, Highsmith Max, Ye Duan, John J. Tanner, Jianlin Cheng

**Affiliations:** 1 Electrical Engineering and Computer Science Department, University of Missouri, Columbia, MO 65211 USA; 2 Departments of Biochemistry and Chemistry, University of Missouri, Columbia, MO 65211-2060 USA; 3 Informatics Institute, University of Missouri, Columbia, MO 65211 USA

**Keywords:** Deep learning, Super clustering, Intensity based clustering (IBC), Micrograph, Cryo-EM, Singe particle pickling, Protein structure determination, AutoCryoPicker, SuperCryoPicker

## Abstract

**Background:**

Cryo-electron microscopy (Cryo-EM) is widely used in the determination of the three-dimensional (3D) structures of macromolecules. Particle picking from 2D micrographs remains a challenging early step in the Cryo-EM pipeline due to the diversity of particle shapes and the extremely low signal-to-noise ratio of micrographs. Because of these issues, significant human intervention is often required to generate a high-quality set of particles for input to the downstream structure determination steps.

**Results:**

Here we propose a fully automated approach (DeepCryoPicker) for single particle picking based on deep learning. It first uses automated unsupervised learning to generate particle training datasets. Then it trains a deep neural network to classify particles automatically. Results indicate that the DeepCryoPicker compares favorably with semi-automated methods such as DeepEM, DeepPicker, and RELION, with the significant advantage of not requiring human intervention.

**Conclusions:**

Our framework combing supervised deep learning classification with automated un-supervised clustering for generating training data provides an effective approach to pick particles in cryo-EM images automatically and accurately.

## Background

Electron tomography is an important technology of 3D molecular structure reconstruction [1]. In cryo-EM, to build a reliable high-resolution 3D reconstruction of protein structures from Cryo-EM images, one must extract hundreds of thousands of single particle images from 2D cryo-electron microscopy [2, 3]. The use of high-energy electrons can result in radiation damage to specimens during imaging and result in extremely noisy micrographs, and consequently, a limited electron dose is preferred [4, 5]. The signal-to-noise-ratio (SNR) of original (2D) micrographs tends to be very low, with noise from a variety of sources including low contrast, particle overlap, ice contamination, and amorphous carbon [6]. Hence, the task of single particle picking is still challenging in some cases [6]. Many different computational methods have been proposed for the semi-automated single particle picking over the past decades. Single particle picking using template-based matching methods are very sensitive to noise [7–13]. Thus, some initial “good references” have to be selected in advance to ensure that those manually selected examples have less noise compared with the other in the same (2D) micrographs. Similarly, the edge-based [14, 15] and feature-based methods [16–18] show a significant reduction in performance since they are sensitive to the lower contrast of the (2D) micrographs [6]. Deep learning methods for single particle picking have been proposed, including EMAN2.21 [18], DeepEM [6], DeepPicker [20], and FasetParticlePicker [21]. These deep learning methods made significant contributions to addressing the particle picking issue. However, there are some unsolved challenges such as lack of a diversified training dataset, high false-positive rate, and the difficulty of dealing with low-SNR micrographs.

Over the past decade, many different computational methods have been proposed for automated and semi-automated single particle picking tasks. These methods are based on different techniques such as template-based matching, edge detection, feature extraction, and conversational computational vision [4]. Recently, Deep Learning has exponentially grown in the field of machine learning [12, 13]. Many Deep Learning algorithms from the field of computer vision and bioinformatics such as [22, 23] use convolutional techniques to extract features from big data via layers in neural networks [12]. Furthermore, deep learning appears to be a suitable approach for cryo-EM image processing as the size and number of the micrographs per data set are continually increasing while the SNR of micrographs remains low [4].

EMAN2.21 [19] proposed to train two CNNs. One for pick particles from the (2D) micrographs while another to distinguish between “good particles” and “bad ones”. For the good and bad references, both should be precisely selected based on two criteria. First, the good training samples “references” should be in pure good particles. Second, the bad training samples are a collection form noisy background references that are selected from the bad noise region in the 2D micrograph in addition to some bad particle references such as large aggregation, ice contamination, or overlap particles.

DeepEM [6] To tackle the problem of the automated free-template particle picking, DeepEM proposed an automated particle recognition using a binary classification approach based on deep CNN learning. DeepEM requires manually select hundreds of particles (selected by humans) to create the training dataset that has both positive and negative examples of each training dataset. Then, using the sliding window to classify the sub-images to particles or background.

DeepPicker [20] proposed a fully automated particle picking approach using other molecules as training data to train the network based on using two CNNs modules (model training and particle picking). DeepPicker considers the absence of training data by suggesting an alternative training scheme called “semi-automated particle picking with an alternative training strategy”. This technique requires a small set of manually user’s selection training dataset (positive and negative particle samples) to train the CNN model and initialize the particle selection process. Then, the trained CNN classifier is used to select particle images from different testing (2D) micrographs that have the same protein molecule shape.

FastParticlePicker [21] proposed a fast-single particle picking in cryo-EM based on the standard approach of the object detection network using fast R-CNN and Caffe. The FastParticlePicker requires to extract the coordinates (upper-left and lower-right corners) of each particle bounding box for every single particle in each individual (2D) micrograph to train the fast R-CNN on good training examples while the rest regions are either a background or bad training examples. Then, cropping the (2D) micrographs with a sliding window and the testing performance relies on the classification network.

The four deep learning methods present a significant contribution to the main particle picking and selection issue. However, there are some challenges that those methods are facing such as lacking diversified training datasets, false-positive numerosity, and low-SNR micrographs accommodation.

First, regularly for the particle picking methods that are based on the alternative molecules as training strategy such as DeepEM [6], there is no sufficient training dataset that is used to train such a model that able to pick different particle picking shapes in different 2D micrographs. Moreover, other previous works such as DeepEM [6] and FastParticlePicker [21] used insufficient and undiversified training datasets in which cannot accommodate very well in noisy data. In addition to that, some training dataset has been manually labeled and select such as FastParticlePicker [20] which an intensive labor work and against the general term of the fully automated approach. Second, all three previous methods rely on a sliding window technique in which generating a numerous number of false-positive particle detection (FP). Third, fully automated single particle picking has to deal with diversified of cryo-EM images. Different micrographs have different challenges like as intensive background details (local aggregates, overlapped particles, background noise fluctuations, carbon-rich areas, and ice contamination), and different levels of low-SNR micrographs. Previous works have not paid enough attention to propose a general framework that deals with different low-SNR micrographs.

To address these issues, we propose a fully automated deep neural network for single particle picking based fully automated training particle-selection using unsupervised learning algorithms. Hence, we propose a fully automated deep neural network for single particle picking based on the fully automated training particle data generation using unsupervised learning algorithms. We use two clustering approaches (regular clustering algorithm using the Intensity-Based Clustering IBC) [24] and super clustering algorithms using the super k-means [25]) to automatically generate training particle datasets for training the deep neural networks. To accommodate the low-SNR cryo-EM images, a general framework of micrograph preprocessing that has been used in both our last two models [24, 25] is applied to improve the quality of the low-SNR micrographs.

The method is tested on cryo-EM images of the Keyhole Limpet Hemocyanin (KLH) [26], Apoferritin [27], 80S ribosome [28], and β-galactosidase [29]. A key feature of our approach is the use of Non-Maximum Suppression (NMS) [30] during the testing phase in order to reduce the number of false-positive particle detections. Overall, the automated DeepCryoPicker improves the performance of particle picking over semi-automated methods such as DeepEM, DeepPicker, and RELION-2 (using referenced-based picking) [31].

## Results

### Micrographs datasets collection

**Fig. 1:**
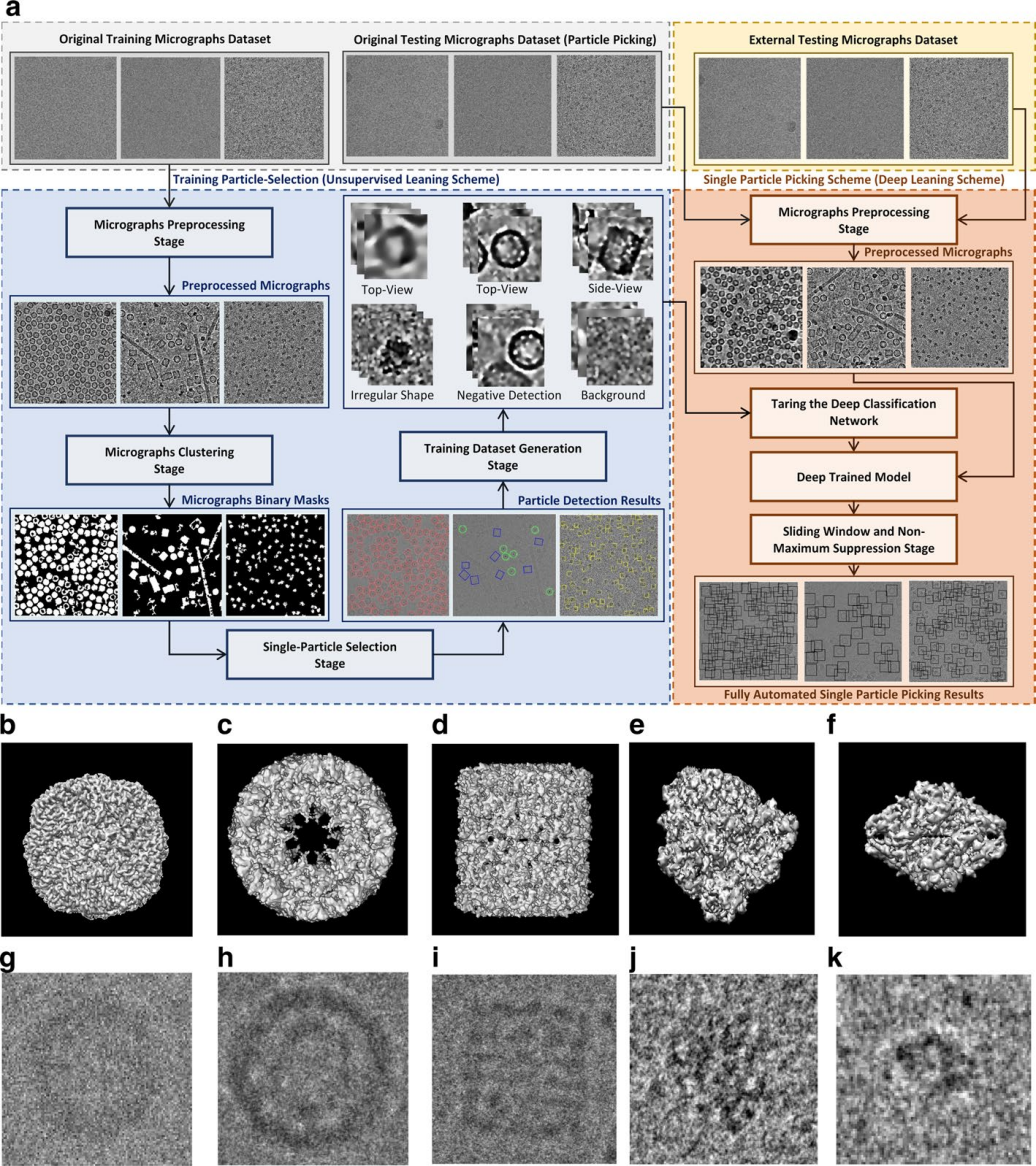
Overview of the DeepCryoPicker procedure. **a** The general workflow of the training particle-selection based unsupervised scheme and single particle picking based on deep learning scheme. The gray part of the workflow shows the micrographs data collection. The blue part of the workflow shows the fully automated training particles-selection using clustering algorithms. The red part of the workflow shows the general flow of the single particle picking using a deep classification network. The yellow part of the workflow shows the external testing part of the DeepCryoPicker. **b** 3D Cryo-EM map of the Apoferritin. **c** Picked particle from an Apoferritin micrograph [27]. **d** 3D Cryo-EM map of KLH is viewed from the top. **e** Picked particle from a KLH micrograph [26] showing the top view (circular particle). **f** 3D Cryo-EM map of KLH is viewed from the side. **g** Picked particle from a KLH micrograph [26] showing the side-view (square particle). **h** 3D Cryo-EM map of the 80S ribosome. **i** Picked particle from a ribosome micrograph [28]. **j** 3D Cryo-EM map of β-galactosidase. **k** Picked particle from a β-galactosidase micrograph [29]

We consider three typical protein shapes in micrographs that are collected from different micrograph datasets as shown in Fig. 1. The first protein shape is circular, as exemplified by the apoferritin [30]. Its 3D Cryo-EM map is shown in Fig. 1b, while a picked particle is shown in Fig. 1c. The second protein shape is square, as seen in the side-view of KLH [26]. There are two main types of projection views in this dataset. The top view is circular (Fig. 1d, e), while the side view is square (Fig. 1f, g). The third protein shape that is considered is the general case of an irregularly shaped protein such as the 80S ribosome (Fig. 1h, i) [28] and β-galactosidase (Fig. 1j, k) [29].

### Performance evaluation metrics

For the evaluation of the performance results we use one of the most popular evaluation metrics which is the precision-recall curve in addition to the accuracy and f1-score [34] that are defined by Eqs. (1), (2), (3), and (4) respectively.

1 $${\text{Precision}} = \frac{TP}{{TP + FP}}$$

2 $${\text{Recall}} = \frac{TP}{{TP + FN}}$$

3 $${\text{Accuracy}} = \frac{TP}{{TP + FP + TN + FN}}$$

4 $${\text{F}}1 - {\text{score}} = 2 \times \left( {\frac{{{\text{Precision }} \times {\text{Recall}}}}{{{\text{Precision }} + {\text{Recall}}}}} \right)$$

where TP is true positives of particles that are correctly picked among the total particles number, FP is the false positives of other objects that are incorrectly detected as particles. FN (false negatives) are particles that are incorrectly predicted as non-particles.

The ground truth labels for the training and testing datasets are manually selected and the number of true-positive (TP) and false-positive (FP) particles are recorded to evaluate the methods.

### Experiments on unsupervised learning framework for fully automated training particles-selection

The automated training particle selection model has two steps: automated training particle picking, and automated training dataset generation. In the first step, 80% of the samples from the collected micrographs are used. Numerous particles are composed and picked from micrograph images using the fully automated framework for particle picking based unsupervised learning approaches that we proposed in our previous models [24, 25]. Then, each single particle image is automatically isolated and evaluated as a “good” or “bad” training sample. The total number of particles for each dataset is shown in Table 1.

**Table 1:** The total number of training particles-selection using fully automated good training particles-selection for apoferritin [27], KLH [26], and Ribosome [29] datasets

Criteria	Apoferritin top-view	KLH top-view	KLH side-view	Ribosome irregular shape	β-Galactosidase complex particle shape
Number of micrographs	20	82		260	84
Training micrograph	10	65		208	67
Testing micrograph	10	17		52	15
Size of micrograph	1240 × 1200	2048 × 2048		4096 × 4096	4096 × 4096
Resolution (Å)	3.1	9.1		3.2	4.2
Voxel size resolution (Å)	0.82	1.24		1.34	1.77
Total number of picked particles	2145	1086	862	5493	8289
Particle’s patch size	178 × 178	221 × 221	221 × 225	187 × 187	214 × 214
Number of good particles	1750	887	689	1076	4781

### Experiments on automated training dataset generation

To address the imbalance problem in the training data, a balanced training dataset is automatically generated. The final training dataset has five classes. Three classes that represent the original particle shapes (top-view, side-view, and irregular (complex) protein shapes) are automatically selected from the “good” particle examples after evaluating every single particle. The image samples of the other two classes are automatically generated from different micrograph’s background as “background class” or automatically expanded and collected from the “bad” training samples as “negative detection class”. Then, a certain number of image samples are randomly selected from each training class to expand the size of the training dataset and generate a balanced training dataset. A sample is rotated 90°, 180°, and 270° to generate three additional training samples. The total number of training particles before and after regeneration is shown in Table 2.

**Table 2:** Automated training particles-selection datasets

Dataset	Before re-generation and selection	After re-generation and selection
Apoferritin top-view	1750	1500
Ribosome Ribosome irregular shape	1157	1500
KLH (top-view)	887	1500
KLH (side-view)	689	1500
β-Galactosidase complex particle shape	4781	1500
Negative detection	–	1500
Background	–	1500

The second column illustrates the total of the particles picked from the training micrographs before applying the good training particles selection and automated training dataset generation and expansion, while the third column illustrates the total number of particles after applying the good training particles selection and automated training dataset generation and expansion

### Experiments on training deep learning classification models

To understand the impact of the number of classes on the classification model, we varied the number of classes in the training dataset via three different experiments. In the first experiment, we used all five classes to train and validate the deep classification model. In the second experiment, we remove the “background” class while keeping the other four classes. In the third experiment, we remove the “negative detection” class while keeping the other four classes. The corresponding precision-recall curve of each experiment in the training dataset showing that in the third case (using three main classes and background class), yields the best result with an average precision of 100%. The average precision is reduced to 98% and 99% in the first and second cases respectively.

### Experiments on testing deep learning classification models

**Fig. 2:**
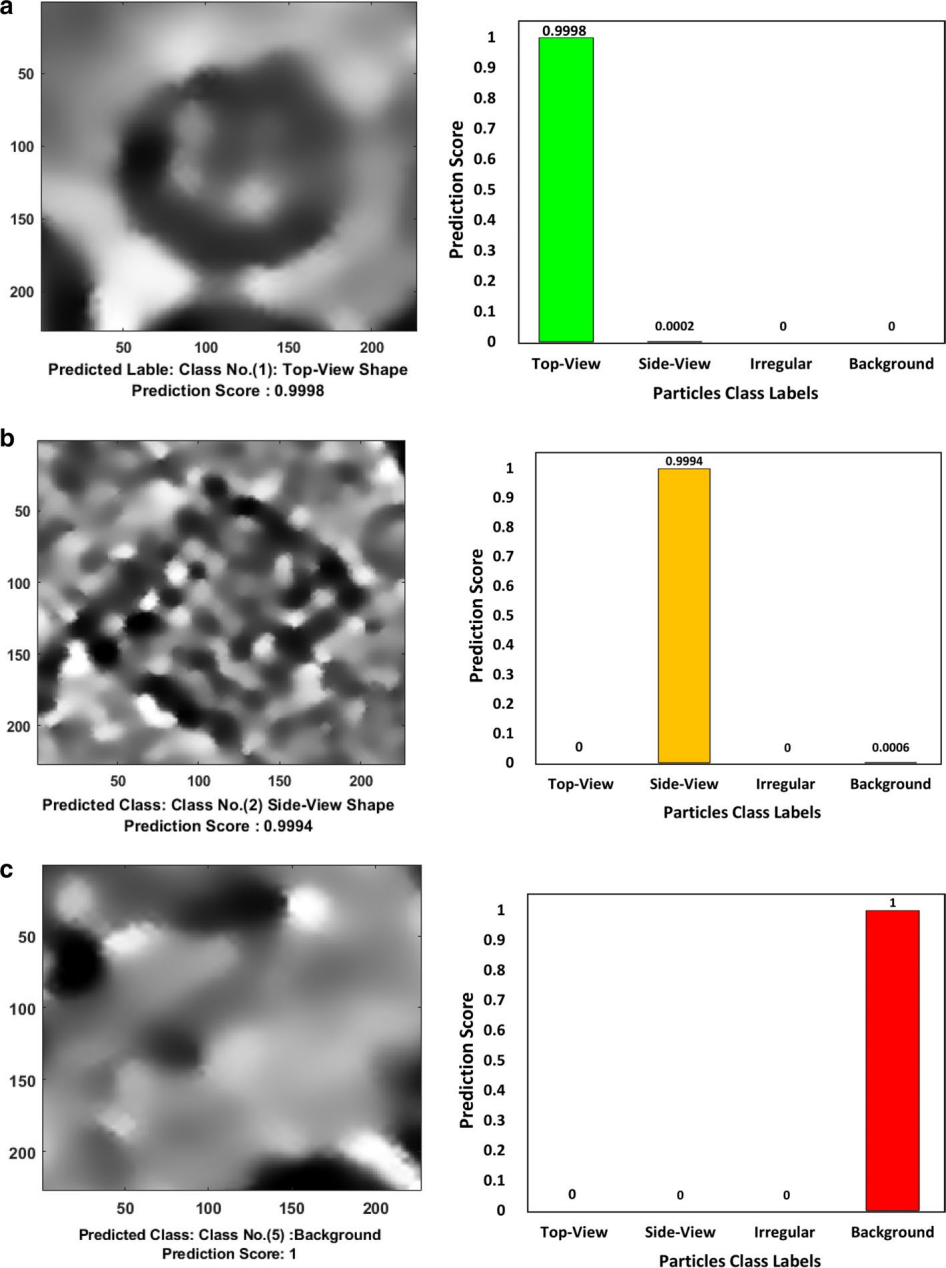

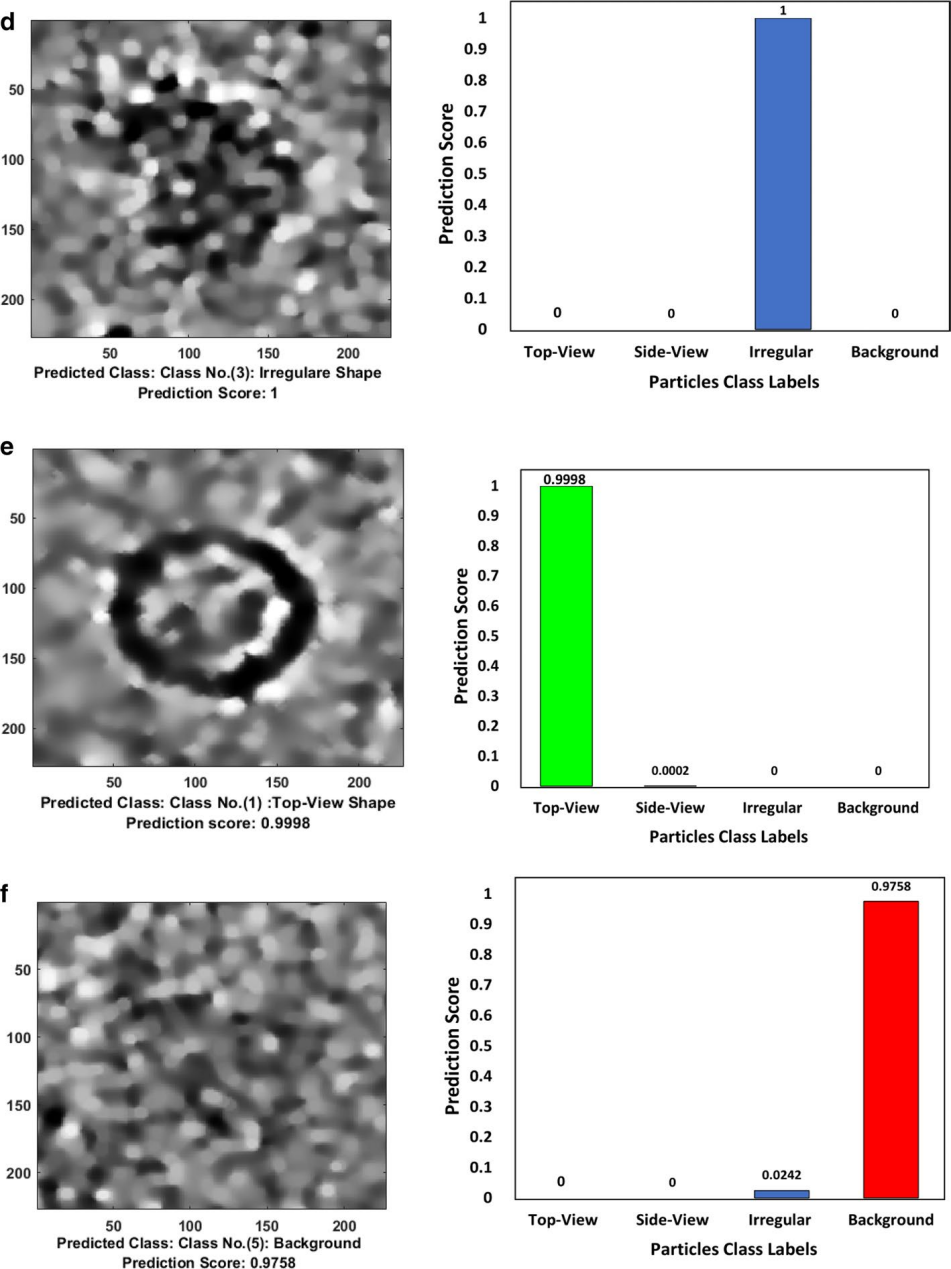
Different examples of the deep classification network results using preprocessed particle images. **a** A typical testing image example showing high-density top-view particle’s predicted label and prediction score of the Apoferritin micrograph dataset [27]. **b** A typical testing image example showing high-density side-view particle’s predicted label and prediction score of the KLH micrograph dataset [26]. **c** A typical testing image example showing high-density background predicted label and prediction score. **d** A typical testing image example showing high-density irregular particle’s predicted label and prediction score of the β-galactosidase dataset [29]. **e** A typical testing image example showing high-density top-view particle’s predicted label and prediction score of the KLH micrograph dataset [26]. **f** A typical testing image example showing high-density background predicted label and prediction score

To evaluate the three deep learning models above, we split our dataset into training, testing, and validation sets. Each class has 1500 particle images, we split the data to 80% for training and validation (1200 particle images, 960 for training and 240 for validation) and 20% testing (300 particle images). The total number of the training particles using 5 classes in the first case is 5250 particles while the total number of the testing particles is 2250. For the second and third models with either background class or negative class, the training set contains 4200 particle images and the testing set contains 1800 particle images. The error or loss of the deep neural network was used as a feedback parameter to tune and adjust the weight and bias, including the number of the feature maps, kernel size of the convolutional layers, and the subsampling kernel size of the subsampling layer. Moreover, the training/testing cycles were tuned based on the hyper-parameters and updated the training datasets until the accuracy of the deep neural network reached a satisfactory level. Figure 2 shows some testing examples of the deep classification network after training based on the third experiment type (three main particle shape classes and background class). The testing accuracy of the deep classification networks using a different number of classes in Table 3. It is clear that the deep classification model achieves a higher accuracy of 99.89% based on using the three-particle classes plus the background cases.

**Table 3:** Test results of using different parameters and datasets

Deep learning model	Learning patch	Epochs	Accuracy (%)
4 class “background”	16	20	99.83
	32		99.89
	64		99.72
4 class “negative”	16	20	97.83
	32		97.78
	64		97.83
5 classes	16	20	96.62
	32		95.96
	64		95.91

Epochs: number of iterations of training

### Experiments on fully automated single particle picking on different micrographs datasets

The second component of our DeepCryoPicker is the fully automated single particle picking. It has three steps: scanning test, scoring cleaning, and filtering using non-maximum suppression. In the first step, a sliding-testing window is used to scan each micrograph in the testing dataset from the top left to the bottom right corner with a constant step size. To determine the prediction parameter, a fixed-size sliding window (square box) is chosen to be slightly larger than the particle size.

The test micrographs have a variety of different dimensions. Before the sliding window, the test micrographs are resized first (scaled up and down) to make sure that the input patches’ coordinates (box size of the sliding window) fit the dimension of the deep network input patch (i.e. 227 × 227).

This step is implemented to automatically use different scaling operations (up-sampling or down-sampling) and different scaling factors. We rely on the two unsupervised models (AutoCryoPicker [24] and SuperCryoEMPicker [22]) to estimate the dimensions of the original particle patches’ coordinates that are detected in the training particles picking and selection stage. First, for each test micrograph, we calculate the average particle patches’ coordinate form each dataset. If the average particle patches’ coordinate is less than the input patch (i.e. 227 × 227), the whole testing micrograph needs to be scaled up. Otherwise, it needs to be scaled down using different factors with a step size of 0.125. The process automatically selects the best scaling factor that gives the best patches’ coordinate that is slightly smaller than the sliding window. Then, the best scaling factor is used to scale the whole micrograph. Finally, the zero-padding operation is used to make the micrograph dimensions to be an even dimension. Table 4 shows the experimental results of the particle patches’ coordinates before and after scaling (resizing) as well as the whole testing micrographs size. For instance, Table 4 (first row) shows the test micrographs from the Apoferritin dataset [27] with dimensions of 1240 × 1200 pixels. Since the average size of the detected top-view particle patches’ coordinates is 94 × 94 pixels, which is less than our input patch size, the testing dataset needs to be scaled up. The best scaling factor of (2.375) is automatically selected which gives the best input patches’ coordinates of 224 × 224 pixels. The test micrographs are scaled up with the same scaling factor to give new testing micrographs dimensions of 3373 × 3373 pixels. Finally, the testing micrographs are unified to be 3374 × 3374 pixels. Table 4 (second and third rows) shows different micrographs from KLH [26] dataset with dimensions of 2048 × 2048 pixels. The average detected top- and side-view particle patches’ coordinates are 187 × 187 and 221 × 221 pixels respectively. In this case, no scaling operation is selected because the smallest scaling factor (1.125) that is applied to scale up the side-views particles gives particle size that is slightly larger than the sliding window (249 × 249 pixels). Also, Table 4 (fourth row) shows different micrographs from the Ribosome dataset [25] with dimensions of 4096 × 4096 pixels. The average detected irregular particle patches’ coordinates are 320 × 320 pixels which the particle image is larger than the sliding window. The best scaling factor that is used to scale down the particle patches’ coordinates is 0.625. That gives the best input patches’ coordinates of 212 × 212 pixels. Then the test micrographs are scaled down to get the new dimension of 4030 × 4030 pixels.

**Table 4:** The results of test micrographs scaling operations

Dataset	Original micrograph dimension	Original particle size	Scaling operation and best factor	Scaled particle size	Scaled micrograph dimensions	Unified micrograph size
Apoferritin top-view	1200 × 1240	94 × 94	Up-sampling (2.375)	224 × 224	3373 × 3373	3374 × 3374
KLH (top-view)	2048 × 2048	187 × 187	No-scaling	187 × 187	2048 × 2048	2048 × 2048
KLH (side-view)	2048 × 2048	221 × 221	No-scaling	221 × 221	2048 × 2048	2048 × 2048
Ribosome Ribosome irregular shape	4096 × 4096	320 × 320	Down-sampling (0.625)	200 × 200	4030 × 4030	4030 × 4030
β-Galactosidase complex particle shape	4096 × 4096	188 × 188	Up-sampling (1.125)	212 × 212	4608 × 4608	4608 × 4608

The second column shows the original test micrographs dimensions, the third column the detected particle patches’ coordinates (dimensions) using AutoCryoPicker [24] and SuperCryoEMPicker [25], the fourth column the selected scaling operation and best factor, the fifth column the scaled particle size using the selected scaling factor, the sixth column the scaled micrograph dimensions, and the last column the unified micrograph size

During the scanning-testing step, every single patch is extracted and fed to the trained deep classification network. Each sliding window receives a certain prediction value [0 1] from the deep network model. The prediction scores represent the probability there is a particle at the center of the corresponding window. In the second step, a scoring map is generated for each tested micrograph. The scoring map describes the likelihood score distribution of the particles over the entire micrograph. In fact, some detected objects such as ice or noise can be predicted as a particle (i.e. false positive). To discard the false positive detection, a cleaning step is implemented which connects any two pixels in scoring maps whose prediction scores are close and both above the threshold. Then, a connected area (pixels) is regarded as a false positive if the size of the connected area is larger than a cutoff value and is removed from the candidate list. Finally, we use non-maximum suppression (NMS) [30] to refine the current particle candidate list. NMS is used to filter the detection boxes based on their Intersection over Union (IoU) between the detected boxes. The candidate particle filtering based on the NMS has three main steps: sorting, selecting and repeating. First, all candidates’ boxes for each given particle category are sorted based on their prediction scores (from high to low). Second, the candidate box that has the highest prediction score is selected as the final candidate box. Then, all other candidate boxes within the selected IoU are discarded. Third, among the remaining boxes, the NMS repeats the two-second steps until there is no remaining box in the candidate list.

**Fig. 3:**
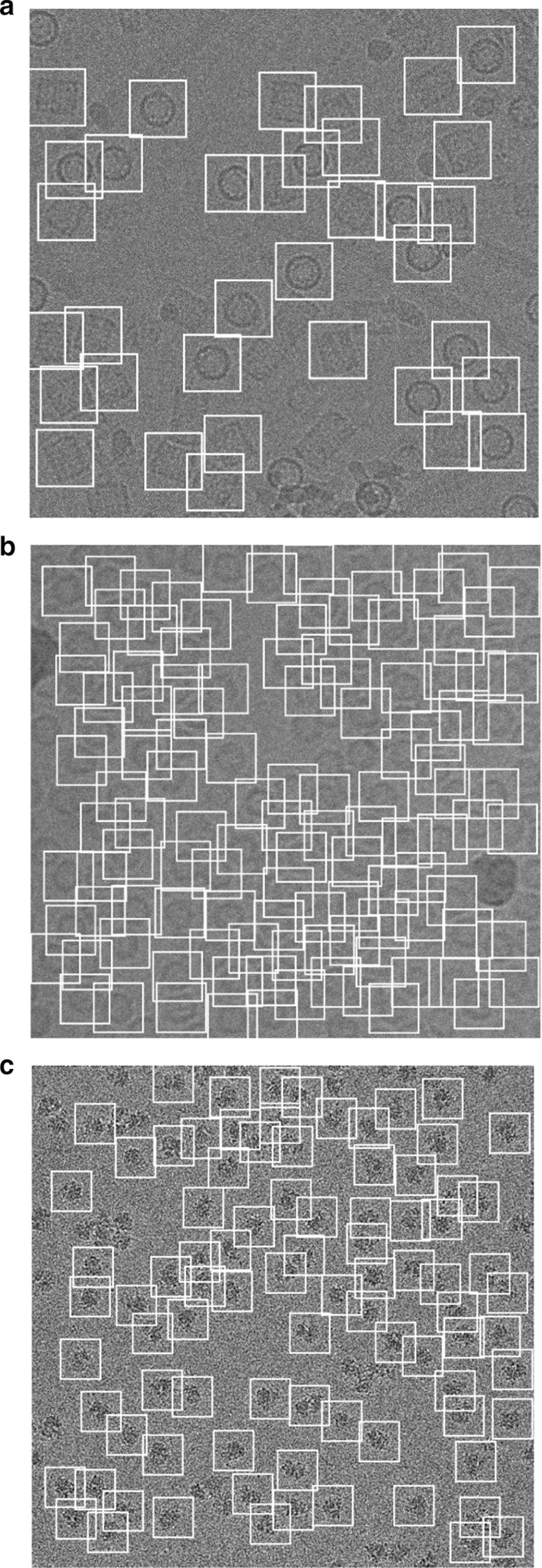
DeepCryoPicker results (different shapes of single particle picking) using three different micrographs. **a** Top and side-view particles picking results using the KLH dataset [26]. **b** Top-view particle picking results using the Apoferritin dataset [27]. **c** Irregular (complex) particle picking results using the Ribosome dataset [28]

**Fig. 4:**
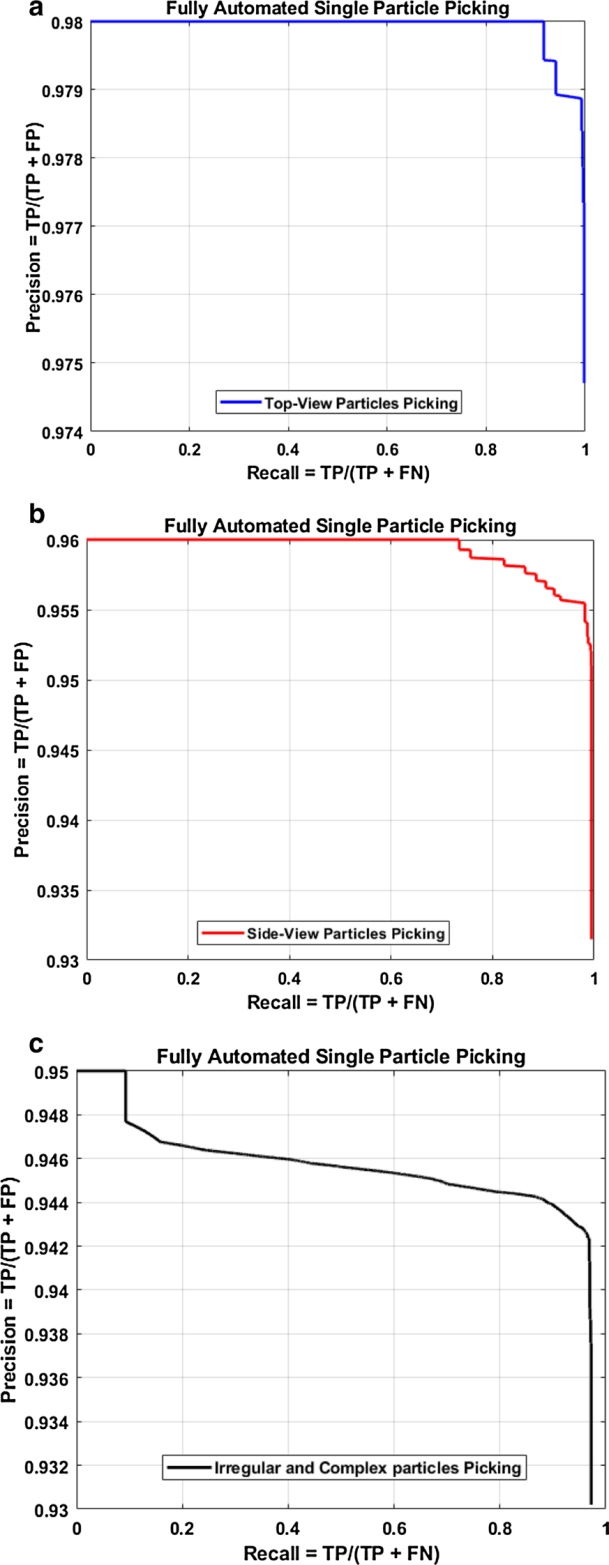
Precision-recall cures of the fully automated different single particle shapes picking result using deep classification network and different micrographs datasets, **a** precision–recall cure of the top-view particle shapes picking. **b** precision–recall cure of the side-view particle shapes picking. **c** precision–recall cure of the irregular and complex particle shape picking

A typical result of DeepCryoPicker is shown in Fig. 3 and Table 5, which illustrates the results of the particle picking using the fully automated framework and different micrographs from different datasets. The average precision-recall reached 97%. Figure 4 shows the precision-recall curves for each particle shapes individually using different datasets such as apoferritin, KLH [26] (the top-view particle shapes), KLH [26] (only the side-view particle shapes), Ribosome and β-galactosidase (irregular and complex particle shapes). For instance, Fig. 4a shows the blue plotted curve of the precession-recall for top-view particle shapes picking, Fig. 4b shows the red plotted curve of the precession-recall for side-view particle shapes picking, and Fig. 4c shows the black plotted curve of the precession-recall for irregular and complex particle shapes picking.

**Table 5:** DeepCryoPicker evaluation table using different micrograph datasets

Trained for	Accuracy	Precision	Recall	F1 score
4 classes with background	0.97	0.94	0.98	0.96
4 classes with negative	0.96	0.99	0.92	0.96
5 classes with background and negative	0.95	0.95	0.88	0.92

Our model is trained for all views (top-view, side-view, and irregular particles) in addition to two optional classes (background and negative samples)

### Experiments on unseen testing micrographs datasets

**Fig. 5:**
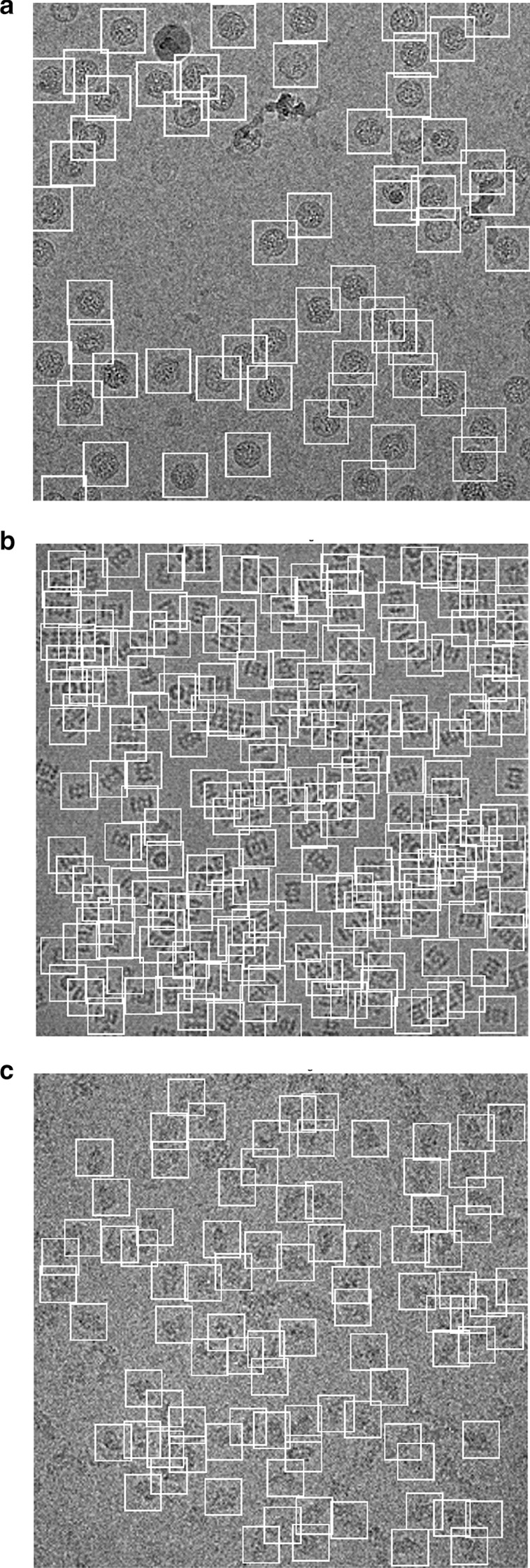
DeepCryoPicker testing results (different shapes of single particle picking) using different micrographs from different external testing datasets (unseen micrographs). **a** Typical external micrograph from the bacteriophage MS2 (EMPIAR-10075) [38] showing the Top-View particles picking. **b** Typical external micrograph from the *T. acidophilum* 20 (EMPIAR-10186) [39] showing the top and side-view particles picking. **c** Typical external micrograph from the β-galactosidase 2.2 ${\varvec{A}}^{^\circ }$(EMPIAR-10061) [40] showing the irregular (complex) particles picking

In addition to testing our model on different test micrographs (testing sets) split from the whole datasets and in terms of the generalization our model to unseen datasets, we further test our model on three different micrographs (external testing micrographs) of other proteins that are different from those of training and test datasets (Fig. 5). The external testing micrographs have been selected based on different particle shapes (Fig. 5a–c). For instance, Fig. 5a is an external testing micrograph from the bacteriophage MS2 (EMPIAR-10075) [38] where the particle shapes are identical top-view. Figure 5b, shows another external testing result on an external testing micrograph from the *T. acidophilum* 20 (EMPIAR-10186) [39] where the particle shapes are either top-view or side-view. Finally, Fig. 5c, shows the last external testing result on an external testing micrograph from β-galactosidase 2.2 Å (EMPIAR-10061) [40] where the particle shapes are irregular.

### Comparing with the state-of-the-art approaches

We compare the results from the DeepCryoPicker with different particle picking tools such as RELION-2 [31], PIXER [4], DeepPicker [20], and DeepEM [6] using the KLH [26] datasets. We evaluate the performance results of our DeepCryoPicker comparing with different particle picking tools such as RELION-2 [31], PIXER [4], DeepPicker [20], and DeepEM [6] based on the precision, recall, accuracy, and f1-score that are defined by Eqs. (1), (2), (3), and (4) respectively.

**Fig. 6:**
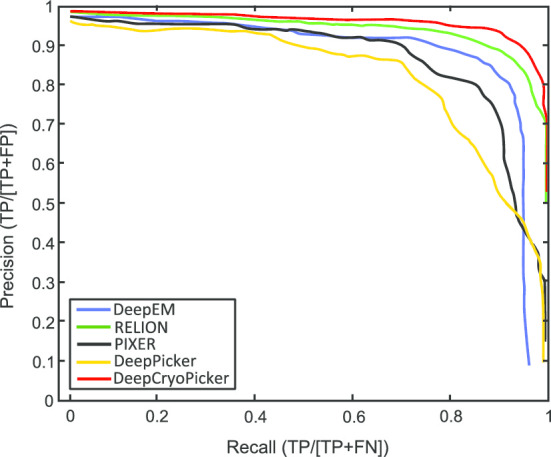
The precision–recall curves of particle picking for different single particle picking tools. The green, yellow, black, blue, and red curves represent the precision-recall curves for RELION-2 [31], DeepPicker [20], DeepEM [6], PIXER [4], and DeepCryoPicker respectively

Figure 6 shows the precision-recall curves of these methods. The blue, green, black, yellow, and red curves represent the precision-recall curves for DeepEM [6], RELION [31], PIXER [4], DeepPicker [20], and DeepCryoPicker respectively. The results indicate that DeepCryoPicker performance is slightly better than RELION-2 [31], with the advantage of being fully automated. The improvement of DeepCryoPicker’s performance over other methods is more pronounced.

In addition to the precision-recall curves, more quantitate analysis of performance different particle picking tools using the popular benchmark KLH [26] are provided. The detailed information on the KLH data [26] that is used to evaluate our DeepCryoPicker with other tools is illustrated in Table 6. First, the whole KLH dataset that has 80 micrographs is divided into 80% of training (60 micrographs) and 20% testing (20 micrographs). Second, each micrograph from the whole dataset is manually labeled from experts and used as a ground truth that contains the number of true-positive (TP) and false-positive (FP) particles. The total number of particles in the training datasets is 1587 (725 top-views and 853 side-views) while the testing dataset has 545 particles (293 top-views and 252 side-views) respectively.

**Table 6:** KLH dataset details used to evaluate our DeepCryoPicker with other particle picking tools

Criteria	Training	Testing
Number of micrographs	60	20
Number of side-view particles	725	293
Number of top-view particles	853	252
Total number of training particle images	1,578	
Total number of testing particle images	545	
Side-view particle size	272 × 272	
Top-view particle size	272 × 272	
Size of micrograph	2048 × 2048	
Single particle reconstruction resolution	2.1 Å	
Voxel size	1.24 Å	

The true particles picking results (TP) among all the total picking results (TP) and false particles picking results (FP) are represented in the precision while the true particles picking results among all the true particle images that the micrograph is contained are represented in the recall. To get a better measure, the F1-score is the harmonic mean of both precession and recall also, the accuracy represents the fraction of all true particles picking among all the classes and the F1-score represents.

The generated precision-recall curve relies on the varying threshold score that each particle picking algorithm used. Threshold balances between both the precision and the recall accordingly. For instance, once the threshold is increased, the precision is increased, and the recall is decreased. In general, the main criteria for any particle picking algorithm at a certain threshold score both precision and the recall are expected to reach higher scores.

For the DeepEM [6] method, we used 1600 particle images (800 positive and 800 negative images) that have randomly selected from the training dataset to train their network. Some parameters need be set such as the particle size that is unified to be 272 × 272 pixels, the classification network’s lower bound is set as a default value 0.6, and the maximum selected number of particles per micrograph. This parameter is set as a default value 500 to help to remove FP particles. For RELION [31] we selected approximately 200 particles to help to generate the template of particles. 100 particle images each top and side-views are manually from the first 10 micrographs. 10 classes are initially selected yielded to 2D classes (side and top-view) templates are low-pass filtered with 20 Å. For PIXER [4], the classification training model includes 5000 particles from different datasets such as 10,017, 10,028, 10,081, 10,097, GroEl, and SIMU. For the classification training dataset, while the segmentation training model includes 10,000 micrographs with 512 × 512 pixels from each training datasets. For DeepPicker [20], the training model includes 10,000 particle images (positive and negative) samples that are manually picked from the TRPV1 training dataset. Both positive and negative samples are normalized into a unified size of 64 × 64 pixels. Validation datasets are separated, the size of each validation dataset is chosen to be 1/9.

**Fig. 7:**
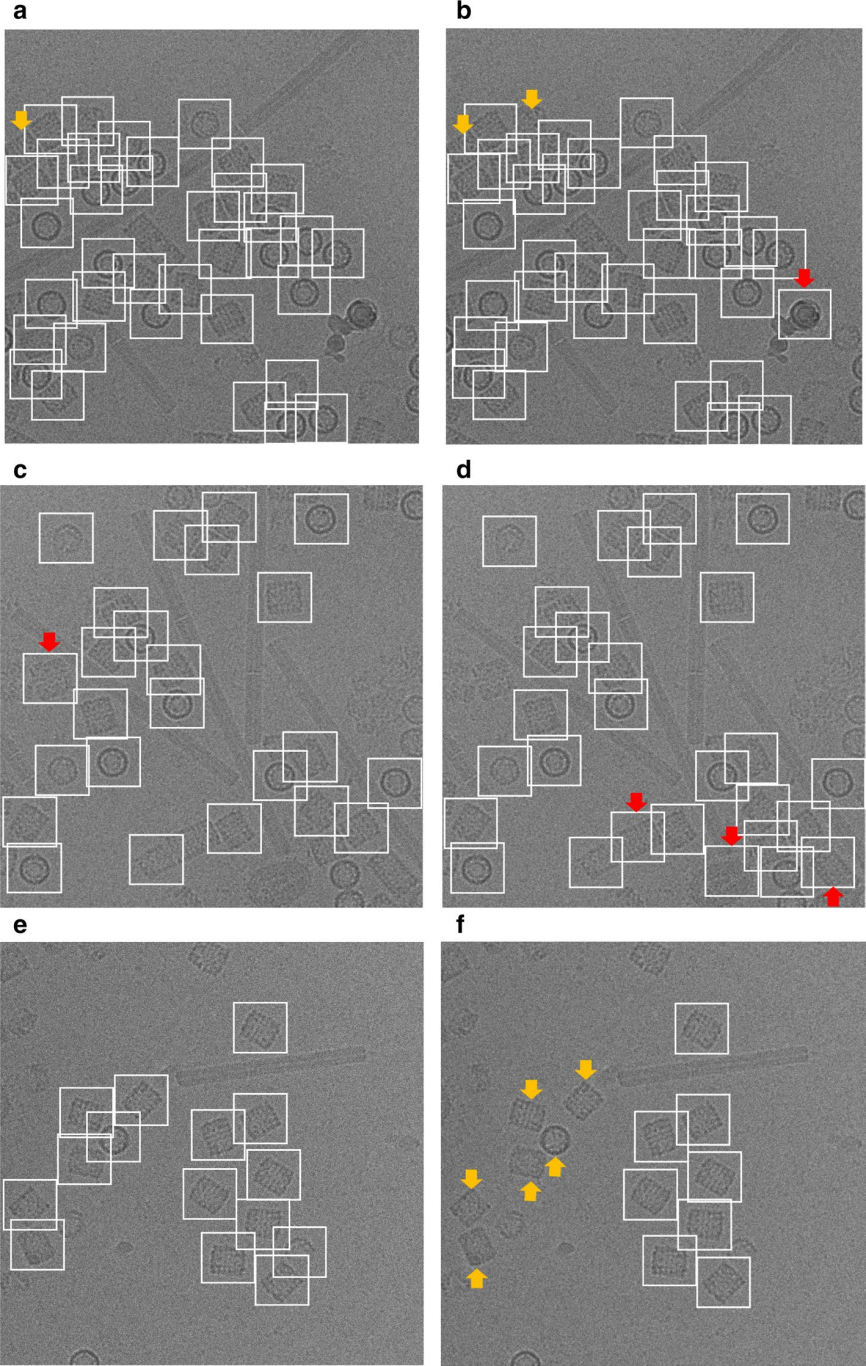

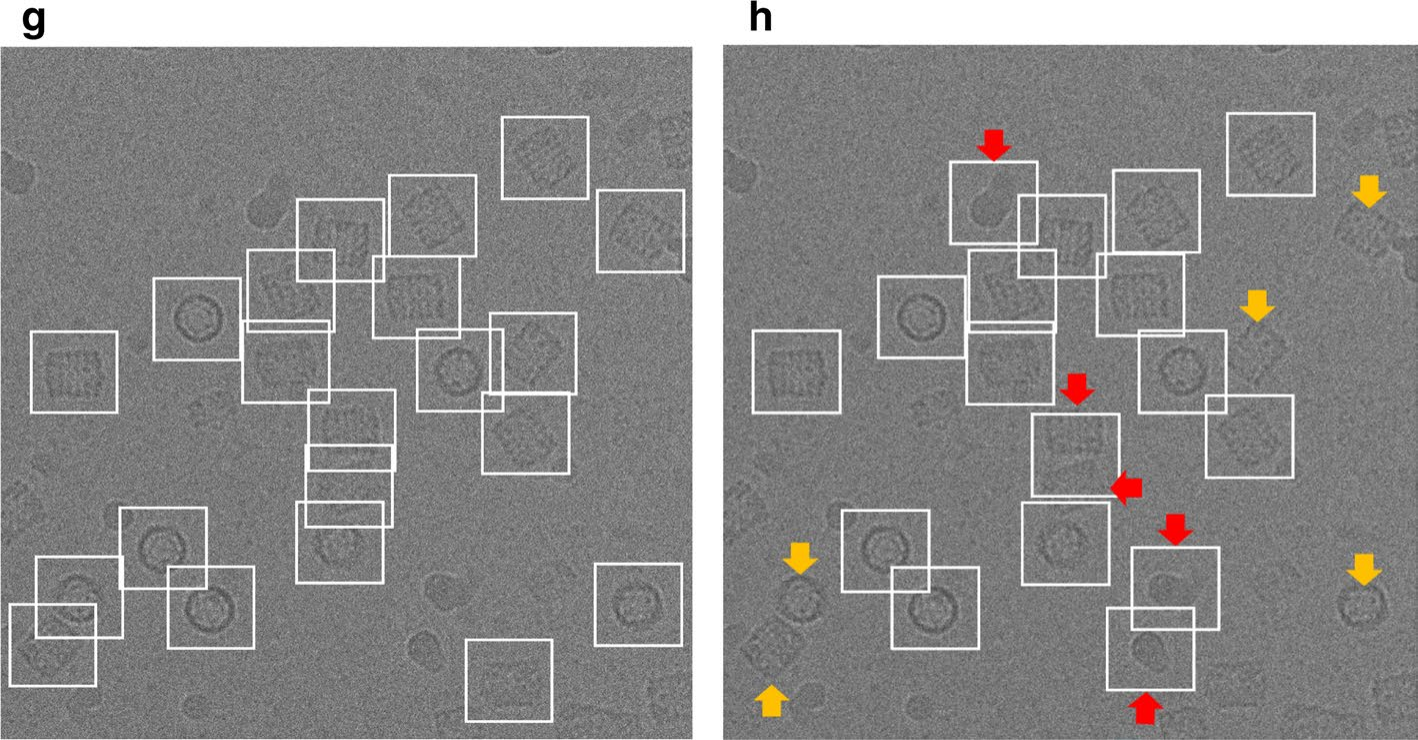
Particle picking results using different testing micrographs from the KLH dataset [26] and different particle picking tools. The red and yellow arrows to denote the FP and FN particles picking results. The red arrows show the FP where the particles are incorrectly picked while the yellow arrows show the FN where some particles are missed (not picked). **a**, **c**, **e**, **g** Top and side-views particles picking results using DeepCryoPicker. **b** Top and side-views particles picking results using RELION [31]. **d** Top and side-views particles picking results using DeepEM [6]. **f** Top and side-views particles picking results using PIXER [4]. **h** Top and side-views particles picking results using DeepPicker [20]

The AURPC values of all compared methods are shown in Table 7. It is noticed that the average precision and recall scores of our DeepCryoPicker is higher than other tools by achieving 94.5% with F1-Score 95.50% while the RELION [31] reach to 94% with F1-Score 93.50%. Also, the average precision and recall scores for DeepEM [6], PIXER [4], and DeepPicker [20] are 89%, 87.5%, and 87.5% with f1-scores 89%, 87%, and 88% respectively. However, for a comparison between our DeepCryoPicker and other tools, Fig. 7 shows the particle picking results using different testing micrographs. Figure 7a, c, e, g illustrate the particle picking results using DeepCryoPicker, while Fig. 7b, d, f, h illustrate the particle picking results using RELION [31], DeepEM [6]. PIXER [4], and DeepPicker [20] respectively. We use red and yellow arrows to denote the FP and FN particles picking results. The red arrows in Fig. 7b, c, d, h show the FP where the particles are incorrectly picked. The yellow arrows in Fig. 7a, b, f, h show the FN where some particles are missed (not picked).

**Table 7:** The AURPC values of all compared methods using the KLH micrographs dataset [26]

Model	Class	Accuracy (%)	Precision	Recall	F1 score
DeepEM	Top-view class	89.04	0.88	0.89	0.88
	Side-view class	89.04	0.9	0.89	0.9
	Average	89.04	89.00%	89.00%	89.00%
RELION	Top-view class	93.98	0.94	0.93	0.93
	Side-view class	93.98	0.94	0.95	0.94
	Average	93.98	94.00%	94.00%	93.50%
PIXER	Top-view class	87.58	0.86	0.88	0.87
	Side-view class	87.58	0.89	0.87	0.87
	Average	87.58	87.50%	87.50%	87.00%
DeepPicker	Top-view class	87.71	0.87	0.86	0.87
	Side-view class	87.71	0.88	0.89	0.89
	Average	87.71	87.50%	87.50%	88.00%
DeepCryoPicker	Top-view class	94.99	0.95	0.94	0.95
	Side-view class	94.99	0.95	0.95	0.96
	Average	94.99	95.00%	94.50%	95.50%

## Discussion

Our method tackles significant challenges that other particle picking approaches have faced such as lack of a diversified training dataset, high false-positive rate, and the difficulty of dealing with low-SNR micrographs. First, to generate such a sufficiently large training dataset, we design a fully automated training particle selection based on unsupervised learning algorithms. Most of the regular protein shapes (circles and squares) have been fully automated picked based on our IBC algorithm. And most of the irregular and complex protein shapes have been accurately picked based on a fully automated unsupervised learning approach using the super k-means clustering algorithm. Therefore, the generation of the training set is fully automated, eliminating the need for manual labeling or labor-intensive particle selection. Second, to accommodate the low-SNR images, a general framework of micrographs preprocessing [23, 24] is applied to improve the quality of the low-SNR micrographs. In general, the preprocessing steps increase the particle’s intensity, and pre-grouping the pixels inside each particle makes it easier to be isolated. Third, to reduce the number of false-positive (FP) particle detections, we use Non-Maximum Suppression (NMS) [30] during the testing phase. It removes duplicates of bounding boxes centered around the same region, consequently decreasing false-positive detections.

### Data augmentation

During the training, instead of passing the original particle image, each image is augmented by using the preprocessed version of the same particle image before passing it through the deep network instead of passing the original image through the network. Each particle patch is modified using different preprocessing methods. During the preprocessing stage, we apply a guided filter operation on the whole micrograph as an edge-preserving smoothing operator. Let us assume that $I$ is a guidance image filter, $p$ is an input micrograph, and $q$ is an output micrograph. Both $I$ and $p$ are given beforehand and can be identical. The filtered output at a pixel $i{ }$ is expressed as a weighted average. We randomly select the SD value between 0 and 1 and then a corresponding filter mask is created. Then the created mask is convolved with the input micrograph using a random mask size selection of 3 and 5. Then, the mask is shifted over the whole micrograph at every single position where the center of the mask is replaced with the output of the guided filter. Also, other methods are applied to the input micrograph such as image normalization to improve the entire contrast between particles and the background. Histogram equalization is used to increase the global image contrast. Image restoration is applied to recover and improve the quality of an image. Adaptive histogram equalization is used to improve the local contrast and enhancing the definitions of edges in each particle. Guided image filtering i performs edge-preserving smoothing of each particle. Morphological image operation is called to enhance the particle shape.

### Computational efficiency

**Fig. 8:**
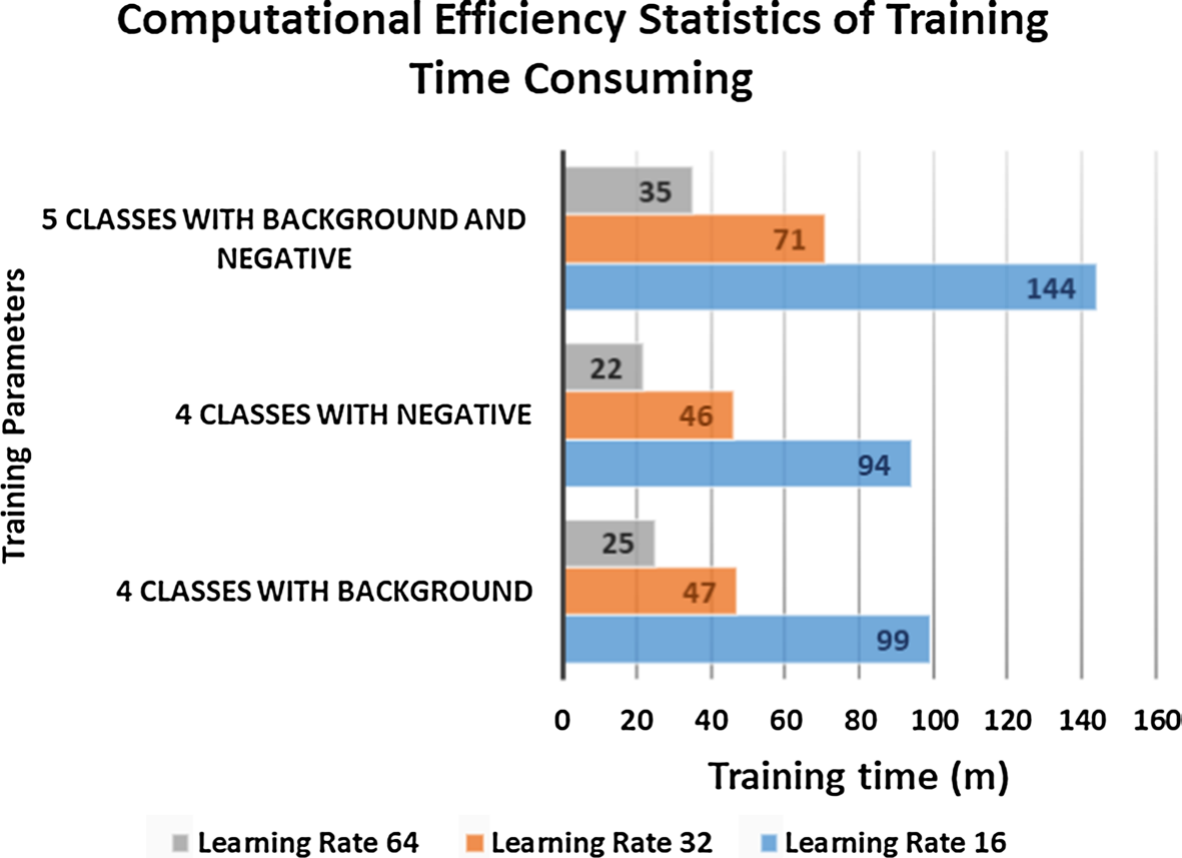
The computational efficiency statistics of DeepCryoPicker training times

We used a desktop computer equipped with an NVIDIA GeForce GTX 1070 graphics card GPU with 4 GB memory and an Intel Core i7 6900 K CPU to train DeepCryoPicker. The time needed for training was 22–144 min for the whole dataset (Fig. 8). The time of particle picking using different hardware systems (GPU and CPU on different micrograph dimensions is shown in Table 8. The average running time per micrograph is 15.7 min on CPU and 1.95 min on a GPU in our experiment.

**Table 8:** Time of particle picking using different hardware systems (GPU and CPU) on different micrograph dimensions

Dataset	Evaluation metric			
	Micrograph dimension	Number of tested micrograph	Testing time (per micrograph) on CPU (min)	Testing time (per micrograph) on GPU (min)
Apoferritin top-view protein shape	3374 × 3374	10	15.8	1.7
KLH (top and side-view) protein shape	2048 × 2048	17	11.3	1.2
Ribosome irregular protein shape	4030 × 4030	52	17.2	2.3
β-Galactosidase complex protein shape	4608 × 4608	15	18.6	2.6

The first column shows the original test micrographs dimensions. The second and third columns illustrate the testing time per micrograph on both CPU and GPU systems respectively

## Conclusions

Our approach for single particle picking from micrographs tackles significant challenges such as lack of training datasets and low SNR micrographs. A manual training datasets preparation (selection and labeling) to train a deep learning approach is a time-consuming and tedious process. Numerous automated single-particle picking based deep learning approaches have been developed and presented a significant contribution of the main particle picking and selection issue. However, there are some challenges that those methods are facing such as lacking diversified training datasets, false-positive numerosity, and low-SNR micrographs accommodation. Here, we present a fully automated deep neural network for single particle picking based self fully automated training datasets generation based unsupervised learning approaches. Our approach solves the fully automated single particle in diversity cryo-EM images when it is tested on real-world datasets from different proteins. The results indicated that DeepCryoPicker performed accurately as good as particle picking state-of-the-art methods.

## Methods

### Overview of the DeepCryoPicker procedure

DeepCryoPicker is designed for fully automated single particle picking in cryo-EM. Our framework contains two components: The first component is a training particle-selection algorithm based on unsupervised learning (shown on the left side of Fig. 1a). The second component is single particle picking utilizing supervised deep learning (shown on the right side of Fig. 1a). The first component has two sections: automated training particles picking, and automated training dataset generation. The first section of the automated training particles selection is based on two steps. Firstly, the micrograph images are pre-processed using a set of advanced image processing tools to enhance and increase the quality of the micrographs. Secondly, each cryo-EM image is clustered using two different unsupervised learning clustering algorithms and then each clustered image is cleaned and used to detect and isolate each particle. Then, some irrelevant objects are removed. The second section of the automated training particle selection is based on automatically evaluating each isolated particle sample and classifying it as a “good” or “bad” training sample. The second component is the fully automated single particle picking method based on a deep learning scheme which has two steps. The first step is designing and training a deep convolutional neural network using the training dataset that has been automatically generated using the first component of our framework. In the second step, the trained model is used to test every micrograph after pre-processing them using the same preprocessing stage that is used to prepare the training dataset. Two different micrograph testing datasets are used for testing.

**Fig. 9:**
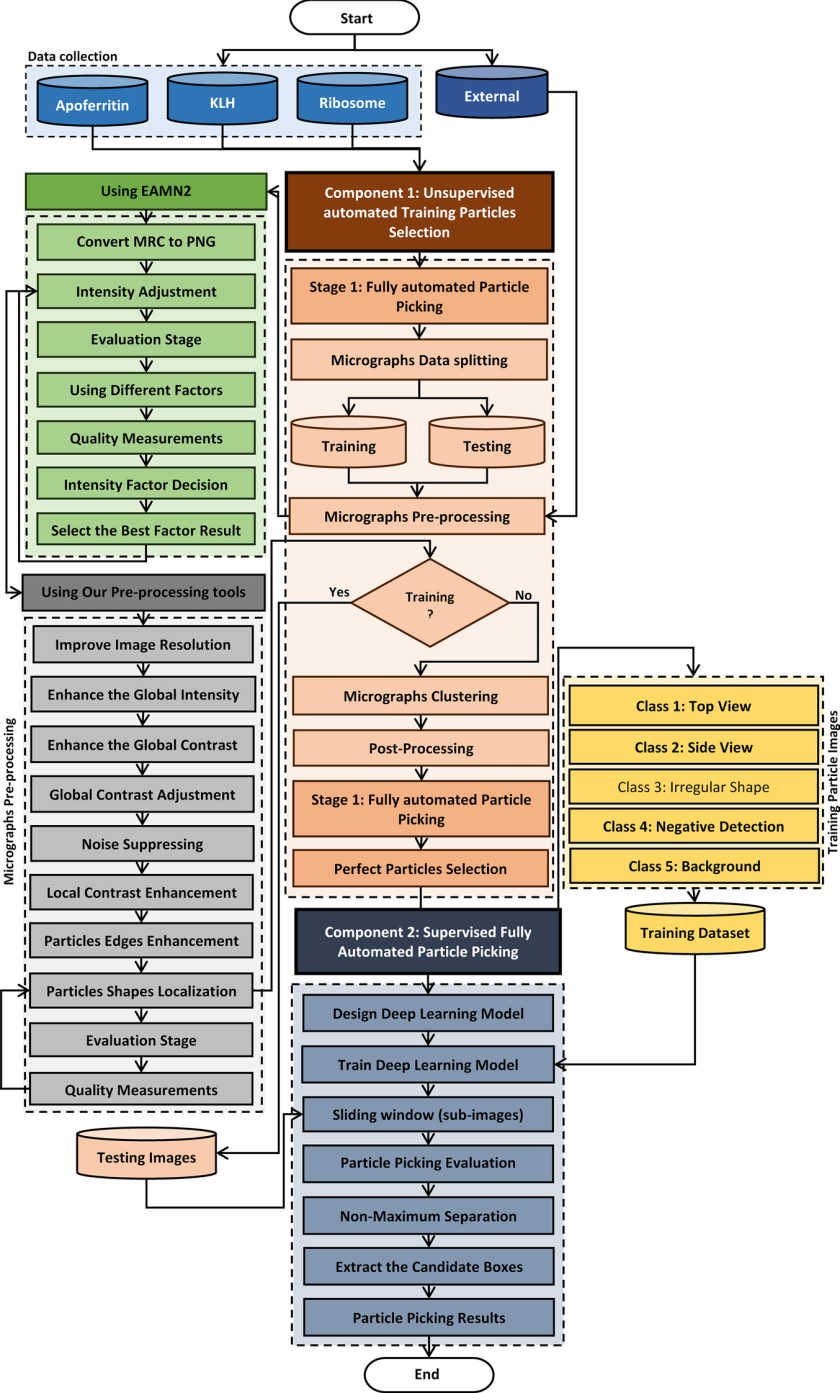
DeepCryoPicker workflow. The orange rectangle marks the first part of the fully automated approach “fully training particles-section and dataset generation”. The blue rectangle marks the second part “fully automated single particles picking”. The green and gray rectangles mark the first and second stages of the preprocessing step respectively

DeepCryoPicker consists of two components (Fig. 9): (1) Component 1: fully automated training particles-selection based on unsupervised learning; (2) Component 2: fully automated single particle picking based on deep classification network. The orange rectangle marks the first part of the fully automated approach “fully training particles-section and dataset generation” while the dark blue rectangle marks the second part “fully automated single particles picking”. The green and gray rectangles mark the first and second stages of the preprocessing step. The blue boxes at the top denotes the datasets used in this work.

### Component 1: fully automated selection of training particles based unsupervised on learning approaches

This component consists of two stages: (1) Stage 1: fully automated training particle selection; (2) Stage 2: fully automated perfect “good” training particle selection and labeled training dataset generation.

### Stage 1: fully automated single particle-picking

**Fig. 10:**
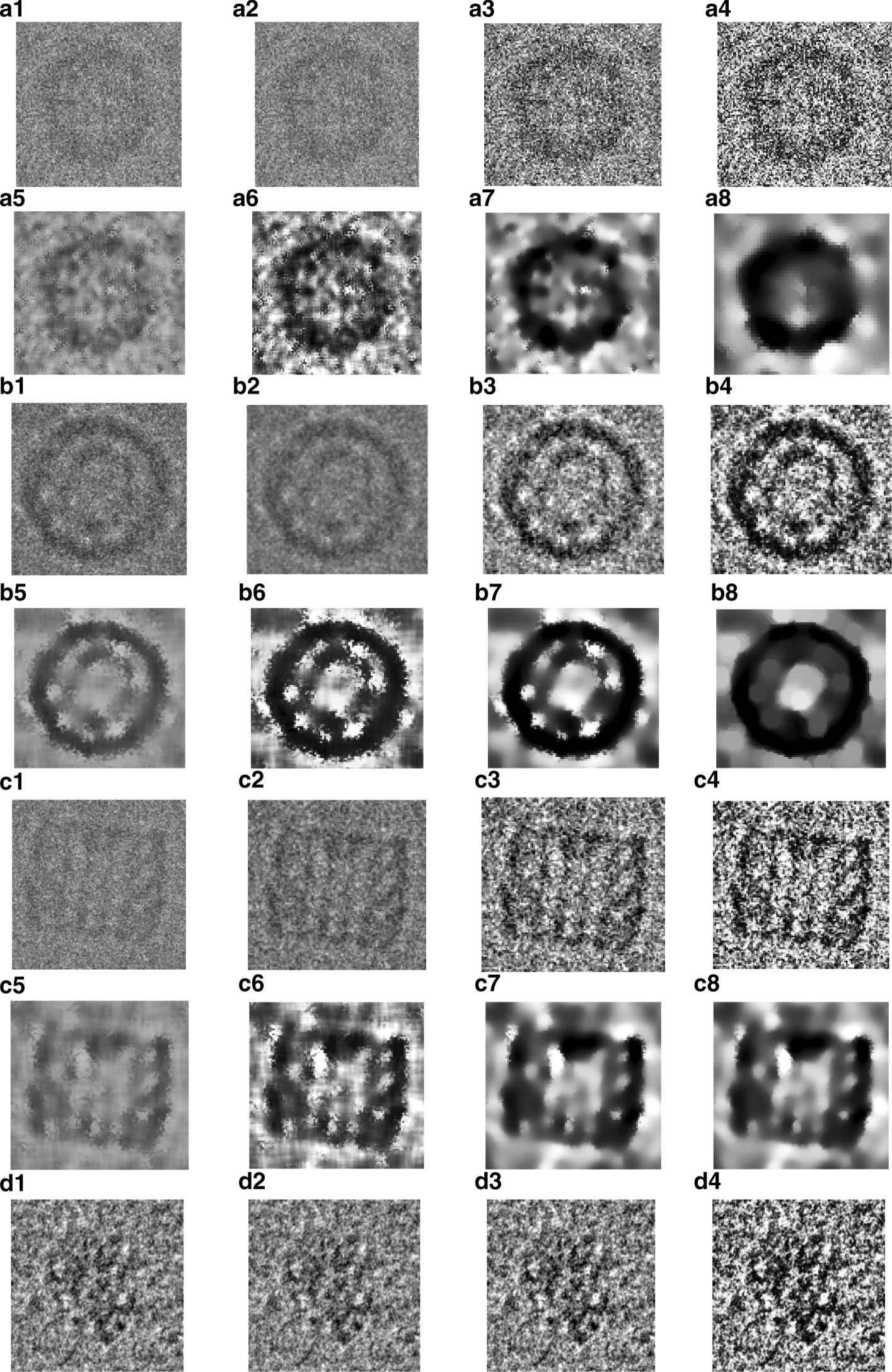

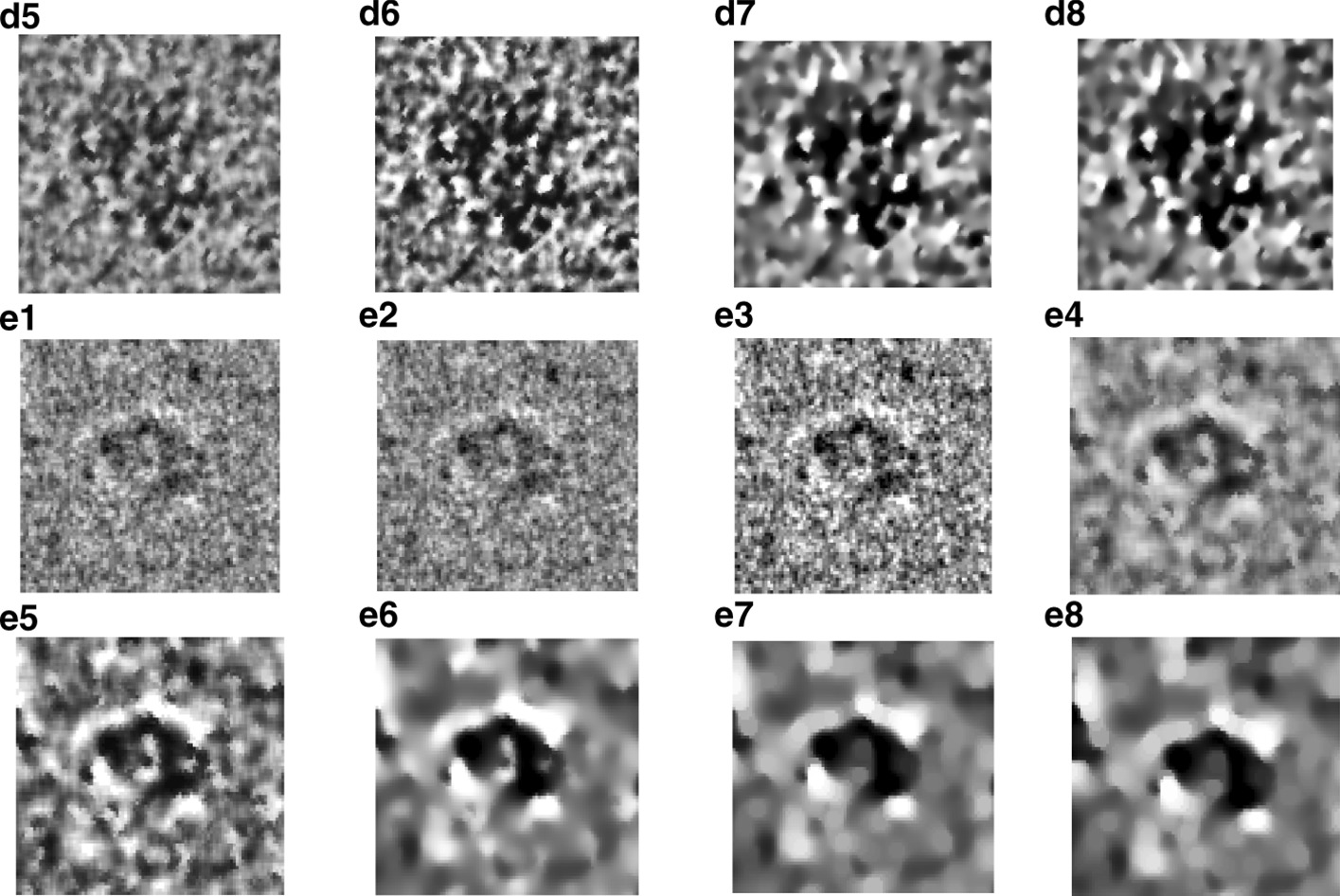
Illustration of the effects of the cryo-EM image analysis on a zoom-in selected particle region using two different examples from two datasets. **a1**, **b1**, **c1**, **d1**, **e1** original zoom-in particle regions (different shapes) are selected from different micrograph Apoferritin (top-view particle) [27], KLH (top-view) [26], KLH (side-view) [26], Ribosome (irregular shape) [28], and β-galactosidase (complex shape) [29] respectively. **a2**, **b2**, **b2**, **e2** normalized single particle image region. **a3**, **b3**, **c3**, **d3**, **e3** single particle region after applying the contrast enhancement correction (CEC). **a4**, **b4**, **c4**, **d4**, **e4** single particle region after applying the histogram equalization. **a5**, **b5**, **c5**, **d5**, **e5** single particle region after applying image resonation with Wiener filtering. **a6**, **b6**, **c6**, **d6**, **e6** single particle region after applying the contrast-limited adaptive histogram equalization. **a7**, **b7**, **c7**, **d7**, **e7** single particle region after applying image guided filtering. **a8**, **b8**, **c8**, **d8**, **e8** single particle region after applying morphological image operation

**Fig. 11:**
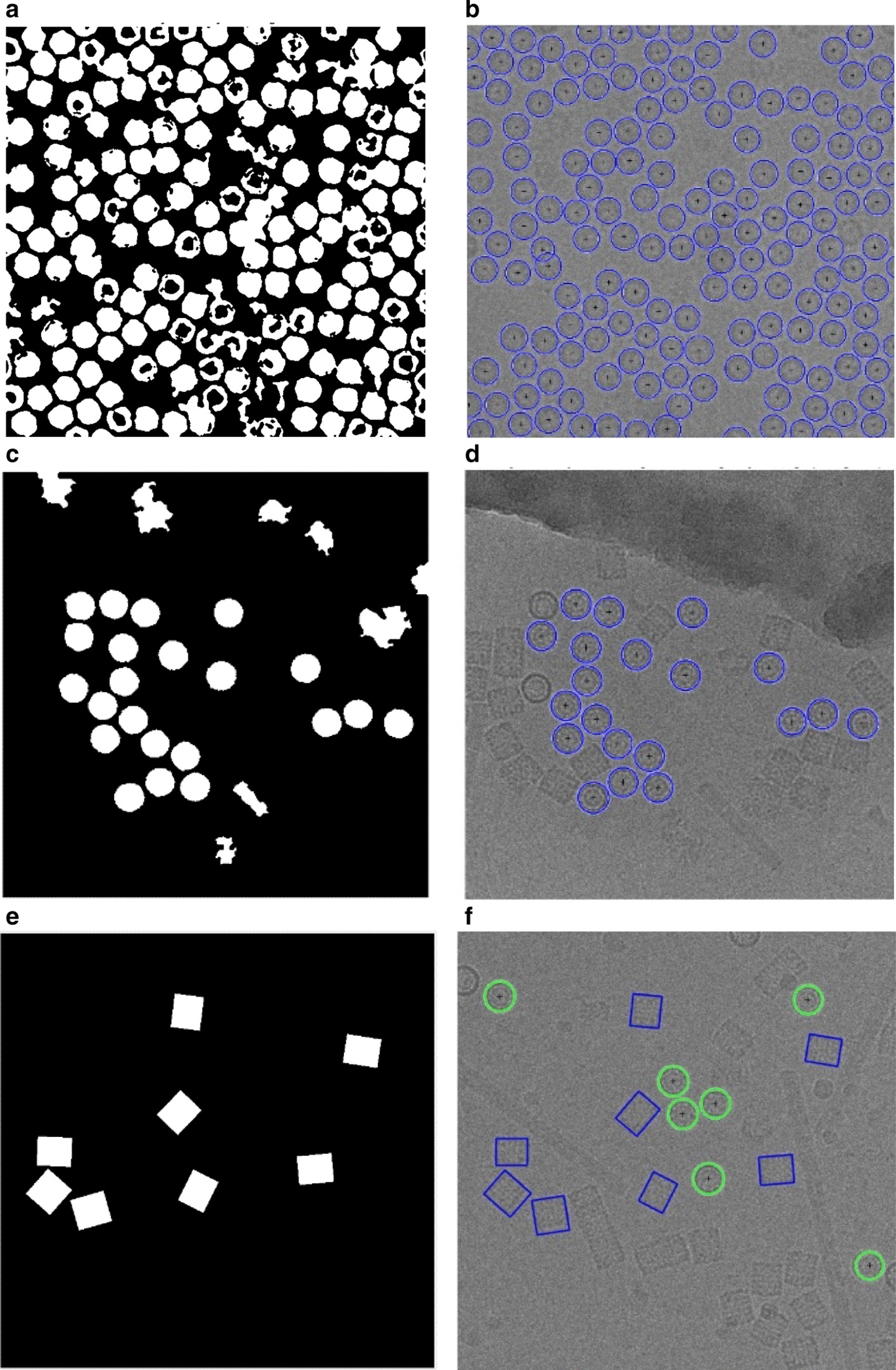

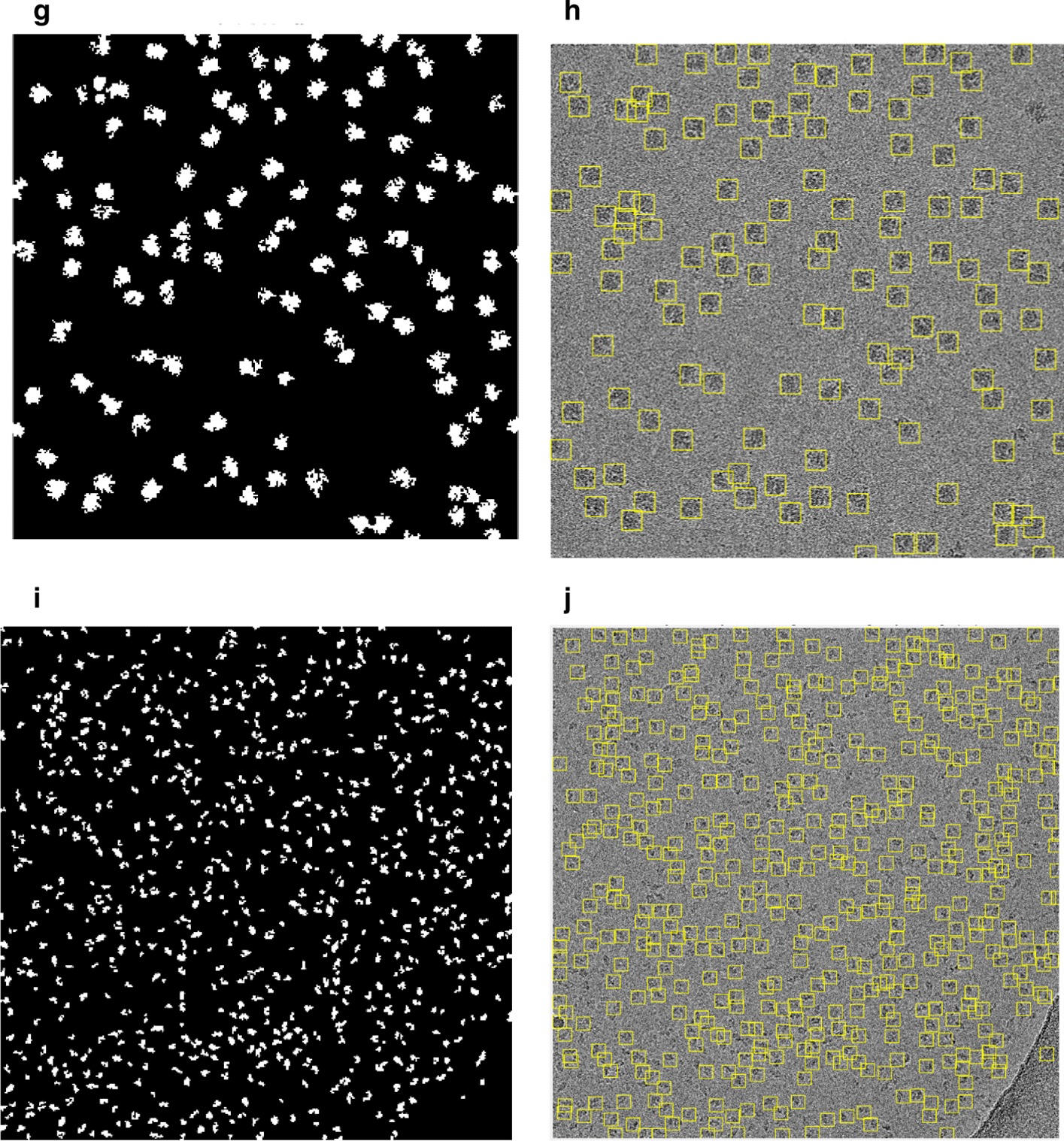
Micrograph clustering and single particle picking results using different cryo-EM datasets. **a** Apoferritin micrograph clustering image (binary mask) using AutoCryoPicker Approach [24] based Intensity-Based Clustering Algorithm (IBC) and Apoferritin dataset [27]. **b** Top-view (Circular) Particles Detection and Picking Results using Modified Circular Hough Transform (CHT) [24], the center of each particle illustrated by the ‘ + ’ sign and the radius of each particle by the blue circle around each particle from the Apoferritin dataset [27]. **c** KLH micrograph clustering image (binary mask) using AutoCryoPicker Approach [24] based Intensity-Based Clustering Algorithm (IBC) and KLH dataset [26]. **d** Top-view (Circular) Particles Detection and Picking Results using Modified Circular Hough Transform (CHT) [24], the center of each particle illustrated by the ‘ + ’ sign and the radius of each particle by the blue circle around each particle from the KLH dataset [26]. **e** KLH micrograph clustering image (binary mask) using AutoCryoPicker Approach [24] based Intensity-Based Clustering Algorithm (IBC) and KLH dataset [26]. **f** Top and side-view (square) Particles Detection and Picking Results using Feret diameters detection [32] and Modified Circular Hough Transform (CHT) [24] from KLH dataset [26], the center of each particle illustrated by the ‘ + ’ sign and the radius of each particle by the blue circle around each particle from the KLH dataset [26]. **g** Ribosome micrograph clustering image (binary mask) using SuperCryoPicker Approach [25] based super k-means clustering (SP-K-means) and Ribosome dataset [28]. **h** Irregular particle shape detection and picking by SP-K-means [25] on the Ribosome dataset [28]. **i** Β-galactosidase micrograph clustering image (binary mask) using SuperCryoPicker Approach [25] based super k-means clustering (SP-K-means) and β-galactosidase dataset [29]. **j** Complex particle shape detection and picking by SP-K-means on the β-galactosidase dataset [29]

Two different fully automated single particle picking approaches based on unsupervised learning (AutCryoPicker [24] and SuperCryoPicker [25]), are used in this stage. AutCryoPicker [24] and SuperCryoPicker [25] used the same preprocessing procedures to increase the SNR and the quality of each micrograph as shown in Fig. 9 (green and gray rectangles). The results of the preprocessing procedures for apoferritin [27], KLH [26], Ribosome [28], and Β-galactosidase [29] images are shown in Fig. 10. The particle picking results that are based on using different unsupervised clustering approaches [24, 25] are shown in Fig. 11.

### Stage 2: fully automated training particle selection

The second stage of the first DeepCryoPicker’s component is the fully automated selection of training particles. After the initial particles are picked and extracted from the first stage “fully automated single particle picking”, each single particle is evaluated to be considered as a good training example using three fully automated perfect “good” training particles-selection approaches such as good top- and side-views particles selection, and irregular/complex training particles selection.

### Perfect “good” top-view training particle selection

We develop an additional step called “good top-view (circular) training particle selection” (see Algorithm 1).
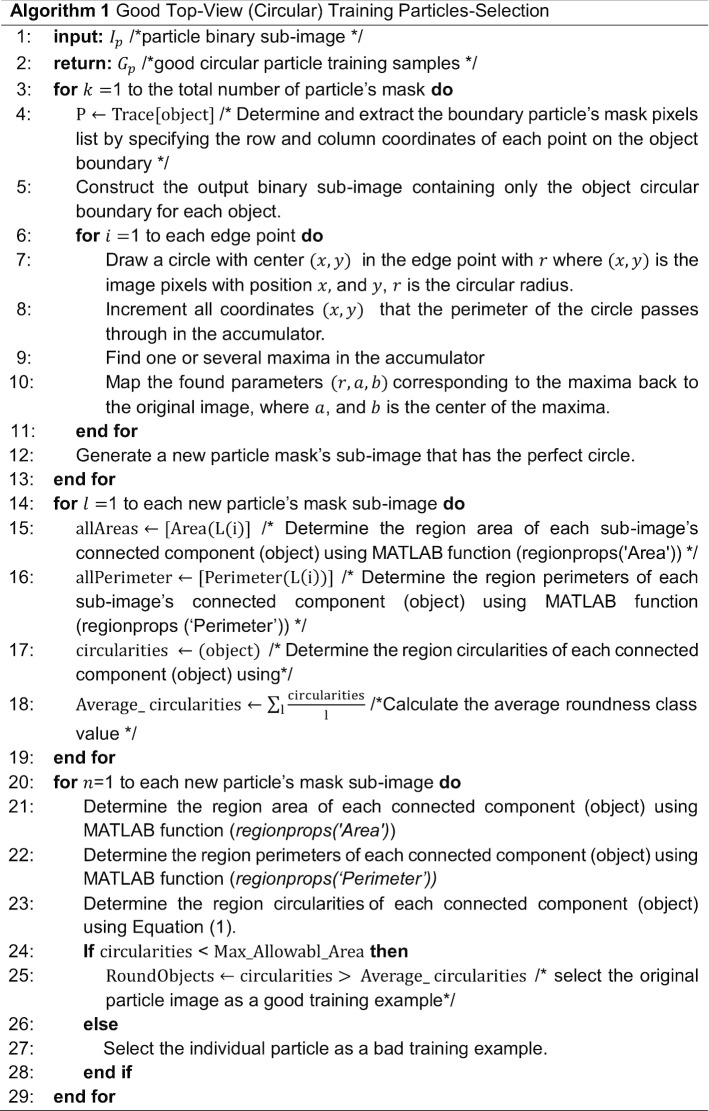


**Fig. 12:**
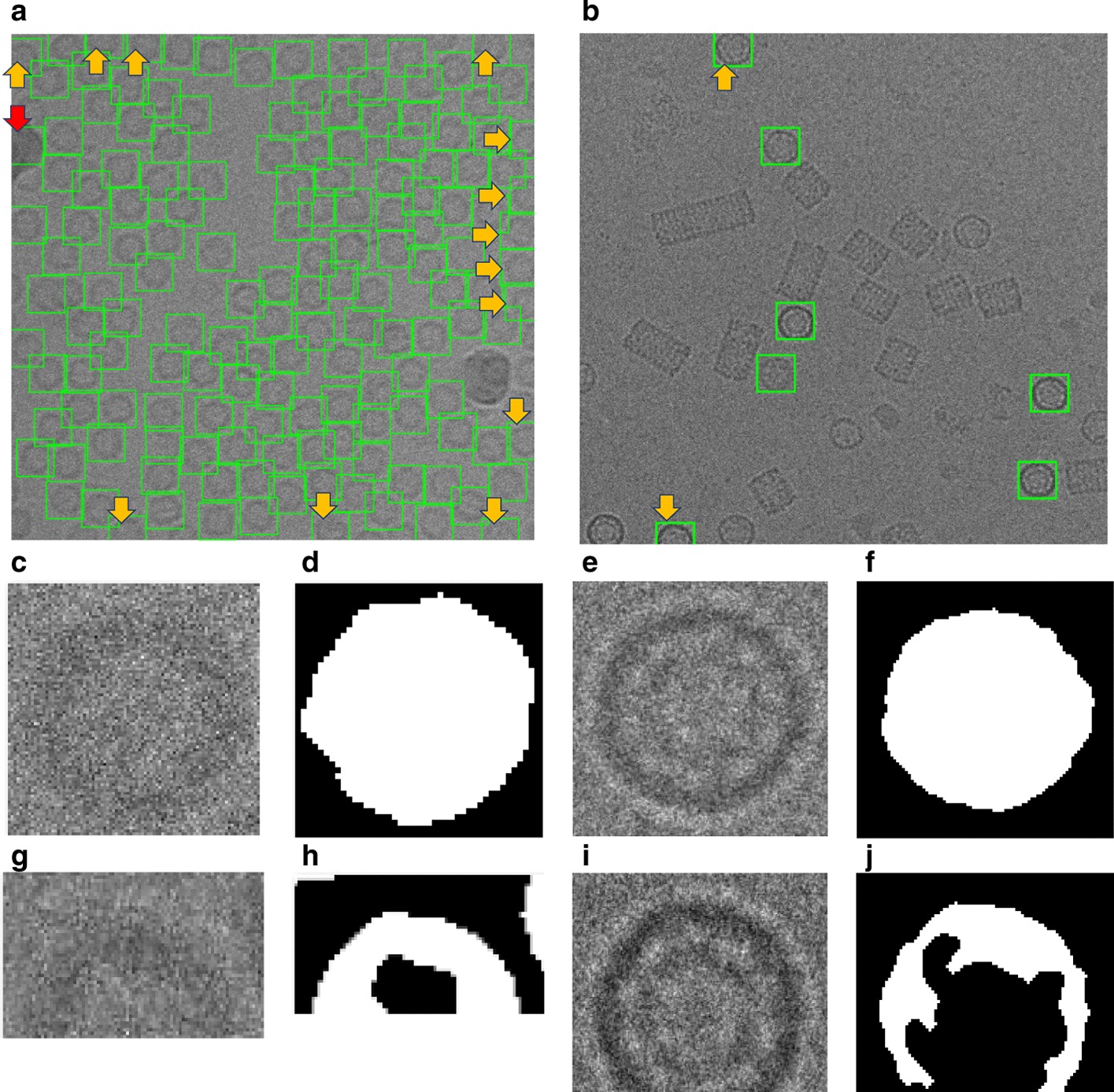
Top-view particles picking results using AutoCryoPicker [24] and different micrographs from the Apoferritin [27] and KLH [26] datasets. **a** Top-view single particle picking results using cryo-EM micrographs form the Apoferritin [27] dataset. **b** Top-view single particle picking results using cryo-EM micrographs form the KLH [26] datasets. **c** Apoferritin good top-view particle example that has been picked using AutoCryoPicker Approach [24]. **d** Apoferritin good top-view binary mask example (perfect “full” binary circular mask). **e** KLH good top-view particle example has been picked using AutoCryoPicker Approach [24]. **f** KLH good top-view mask example (perfect “full” binary circular mask). **g** Apoferritin bad top-view particle example has been picked using AutoCryoPicker Approach [24]. **h** Apoferritin bad top-view binary mask example (non-perfect binary circular mask). **i** KLH bad top-view particle example has been picked using AutoCryoPicker Approach [24]. **j** KLH bad top-view binary mask example (non-perfect binary circular mask)

**Fig. 13:**
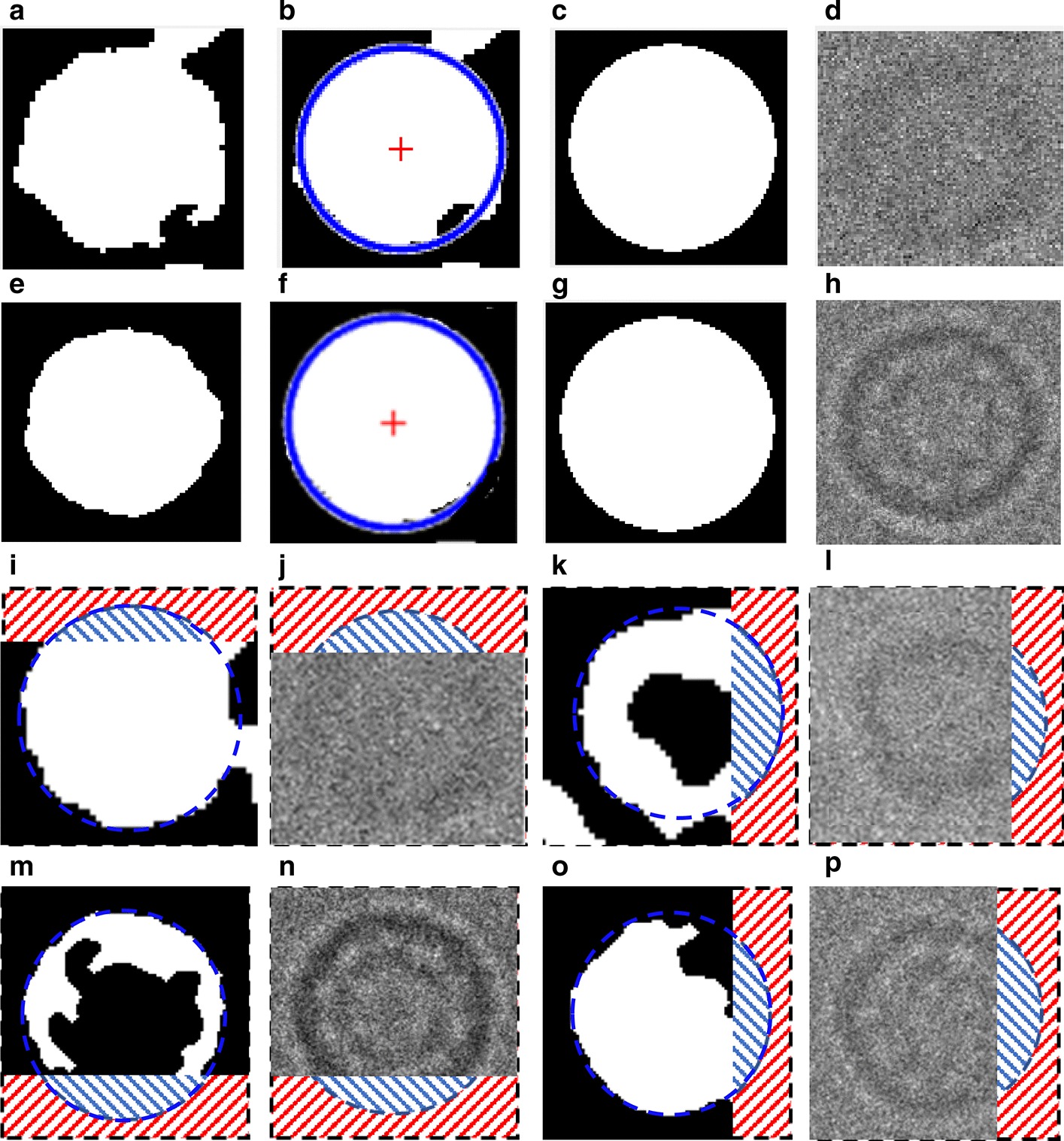
Fully automated good training top-view training particles-selection results using AutoCryoPicker [24] approach and diferenrt micrographs from Apoferritin [27] and KLH [26] datasets. **a**, **e** Individual top-view particle binary mask form the Apoferritin [27] and KLH [26] datasets. **b**, **f** CHT [24] perfect circle on top of the particle’s binary masks. **c**, **g** Generated perfect top-view binary mask based on the center and dimeter that are automatically extracted from the CHT [24] using picked top-view particles form Apoferritin [27] and KLH [26]. **d**, **h** The full automated good top-view training particle selection results based on the perfect mask generation using CHT [24] and different top-view picked particles from different datasets (Apoferritin [27] and KLH [26]). **i**, **k**, **m**, **o** Other examples of the top-view particle’s binary masks that the modified CHT [24] has failed to draw perfect circles on top of them (dash red lines illustrate the missing part of the particle’s background while the dash blue lines illustrate the missing part of the circular object). **j**, **l**, **n**, **p** The full automated bad top-view training particle selection using different top-view picked particles from different datasets (Apoferritin [27] and KLH [26]) (dash red lines illustrate the missing part of the particle’s background while the dash blue lines illustrate the missing part of the circular object)

This step is based on using the individual binary mask for each particle as shown in Fig. 12d, f, h, j. Then, we use the modified Circular Hough Transform algorithm (CHT) in AutoCryoPicker [23] to generate a perfect circle on top of each particle’s mask. Then, we test each individual particle’s mask size and verify if it is a perfect full circle and label it as either a “good example” or as a “bad example”. We test each top-view particle by calculating the average roundness value for the whole top-view (circular) particles. This is determined by computing the area and perimeters using the connected component particle mask’s pixel index list and the circularity based on the Eq. (5):

5 $$Circularities = \frac{{allPerimeters^{2} }}{4 \times pi \times allAreas}$$

where $allAreas$ is the area of each selected particle and $allPerimeters$ is the cemetery size of each particle. Then, each individual particle (circular) does achieve the average object roundness class is considered as a “good” training example, otherwise as a “bad” training example. Figure 13 shows the results of the good top-view training particle selection. Figure 13a and e show individual top-view particle binary masks from the apoferritin [27] and KLH [26] datasets. It is noticed that a perfect circle has been successfully drawn on top of the particle’s binary mask using the modified CHT algorithm as shown in Fig. 13b, f. Figure 13c, g show the replaced artificial perfect circle binary masks that will be used later to test the particles for apoferritin [27] and KLH [26] datasets. Figure 13d, h show the good apoferritin [27] and KLH [26] top-view training particles selection. In contrast, Fig. 13i, l, m, o show other examples of the top-view particle’s binary masks that the modified CHT has failed to draw perfect circles on top of them. Figure 13j, l, n, p show some bad top-view training particle examples.

### Perfect “good” side-view training particles-selection

**Fig. 14:**
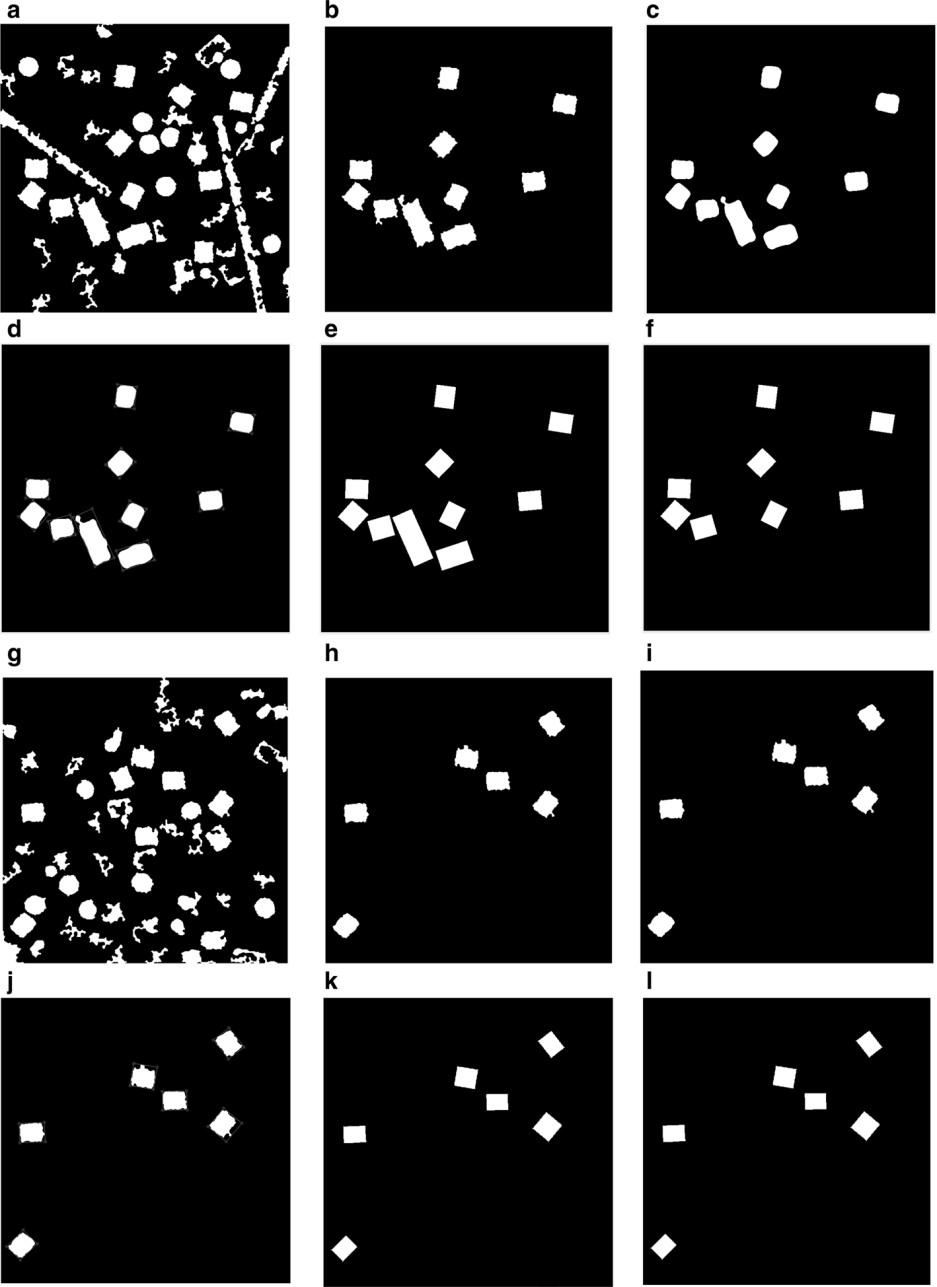
Fully automated side-view particles clustering results using different cryo-EM micrographs and Intensity-Based Clustering Algorithm (ICB) [24]. **a**, **g** KLH micrograph clustering images (binary masks) using the KLH dataset [26] were both top and side-view particles appear in additional to some cumulative ice and artificial objects. **b**, **h** Cleaned KLH micrograph binary mask images that have only the side-view particles after micrograph cleaning and small object and circular objects removal. **c**, **i** Binary particle objects smoothing micrographs. **d**, **j** Feret diameters measures [32] for the particle objects. **e**, **k** Perfect side-view (square) particle shapes generation on the top of the binary image of the KLH micrograph. **f**, **l** Show the overlapped particles removal and perfect side-view particles-selection results after remove the overlapped side-view binary masks

**Fig. 15:**
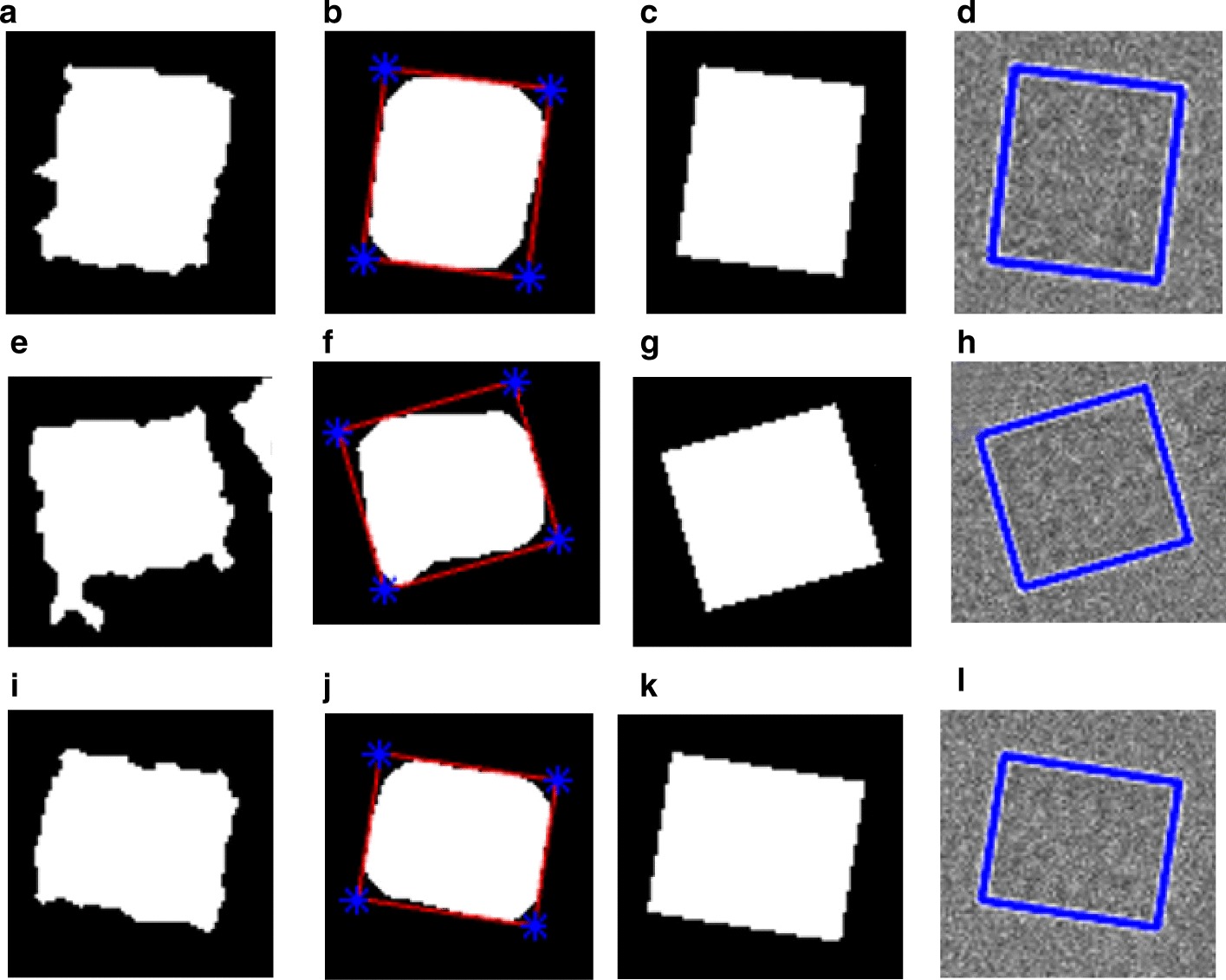
Fully automated perfect side-view masks generation and good training particles-selection results using the KLH dataset [26]. **a**, **e**, **i** The original individual side-view particle binary masks. **b**, **f**, **j** New binary particle’s mask dimensions using Feret diameters [32]. **c**, **g**, **k** The replaced artificial perfect side-view (square) binary masks based on the new Feret object dimensions. **d**, **h**, **l** The good KLH side-view particles selection

**Fig. 16:**
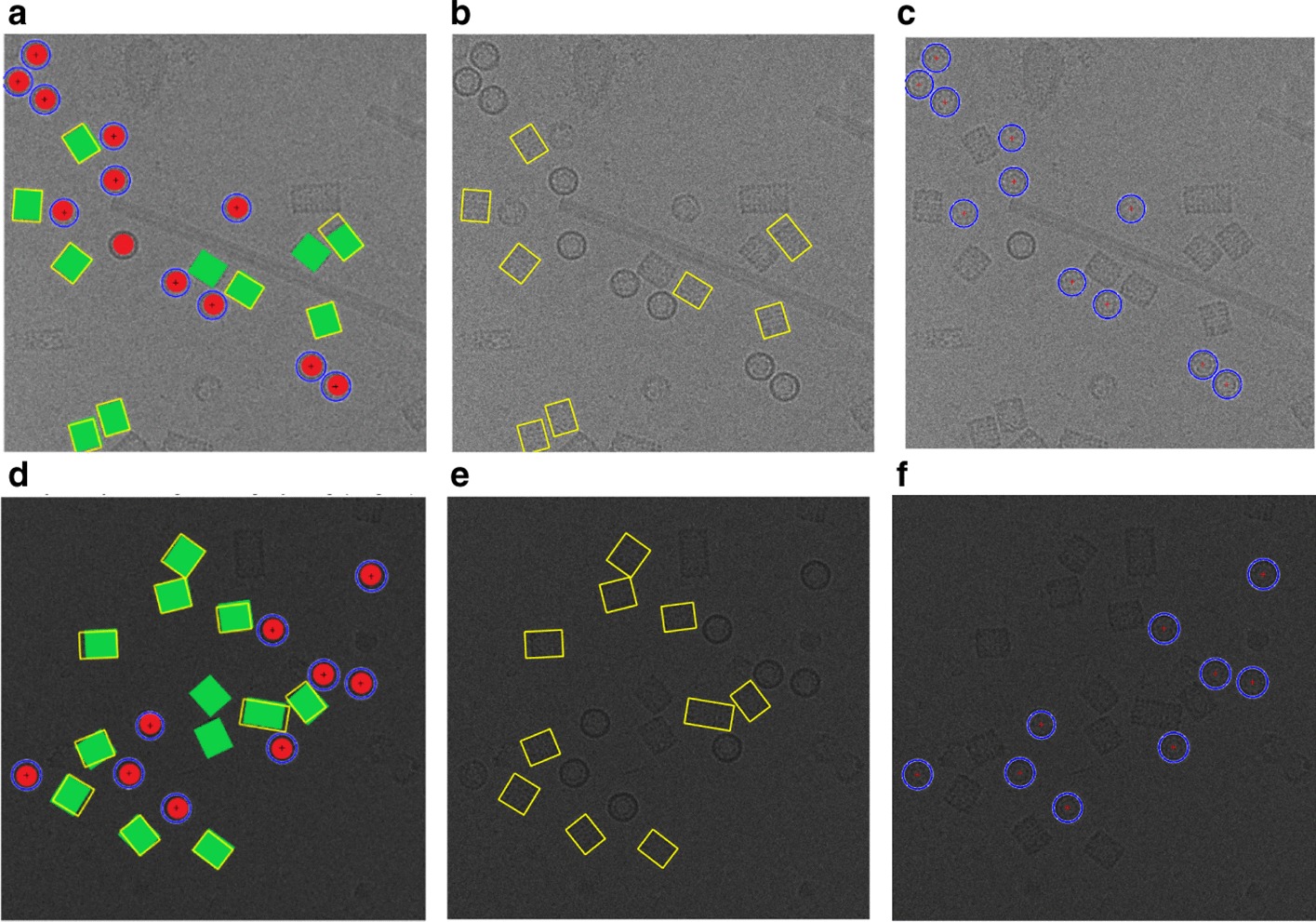
Fully automated good top and side-View (square and circular) training particles-selection using AutoCryoPicker [24] approach and KLH dataset [26]. **a**, **d** The Ground truth (particles manually labeled) for the different cryo-EM images from the KLH dataset [26]. **b**, **e** Side-view particles-selection results using AutoCryoPicker based IBC algorithm [24] and perfect side-view (square) particles-selection algorithm. **c**, **f** Top-view particles-selection results using a modified CHT algorithm [24] (the red ‘ + ’ sign is the center of each particle, and blue circles around each particle are the radius of each particle by the blue circle around each particle

For the side-view particles picking, we do not have an issue with the overlapped particle selection since the only perfect side-view (square) particles are selected through the side view (square) training particle shape selection in cryo-EM based on using the “overlapped particles removal and perfect side-View particles selection algorithm” in the AutoCryoPicker [24]. Figure 14a, g show different KLH cryo-EM clustering results using the Intensity-Based Clustering Algorithm (ICB). Figure 14b, h show the KLH cryo-EM clustered images after the circular and non-square object removal. The binary mask images have only the square particle shapes (side view) in the whole cryo-EM images. Some overlapped particles still exist in the cleaned binary mask as is shown in Fig. 14b. The overlapped particles are removed from the final cleaned masks (See Fig. 14e, f) after applying the overlapped particles removal using the Feret diameter measures approach [32] (see Fig. 14d, j). Figure 14f, l show the same KLH binary mask images after the perfect side-view (square) particles shape generation is applied to the cleaned binary masks. Figure 15 shows an example of the perfect side-view (square) particle selection. Figure 15a, e, i illustrates the individual side-view particle binary masks, while Fig. 15b, f, j show the new binary particle’s mask dimensions using Feret diameters [32]. Figure 15c, g, k show the artificial perfect side-view (square) binary masks based on the new Feret object dimensions. Finally, Fig. 15d, h, l depict the good KLH side-view particles selection. Figure 16a, d show top and side-view particles picking using different cryo-EM micrographs form the KLH dataset. Figure 16b, e show the final results of side-view particles-selection using different micrographs form the KLH dataset based ICB clustering, and perfect square (side view) particle shape detection using Feret object diameter [32]. Figure 16c, f also show the top-view particles-selection results based on modified ICB clustering and modified CHT [21]. Also, Fig. 16a, d show the ground truth of both top and side-view particles.

### Perfect “good” irregular and complex training particle-selection

**Fig. 17:**
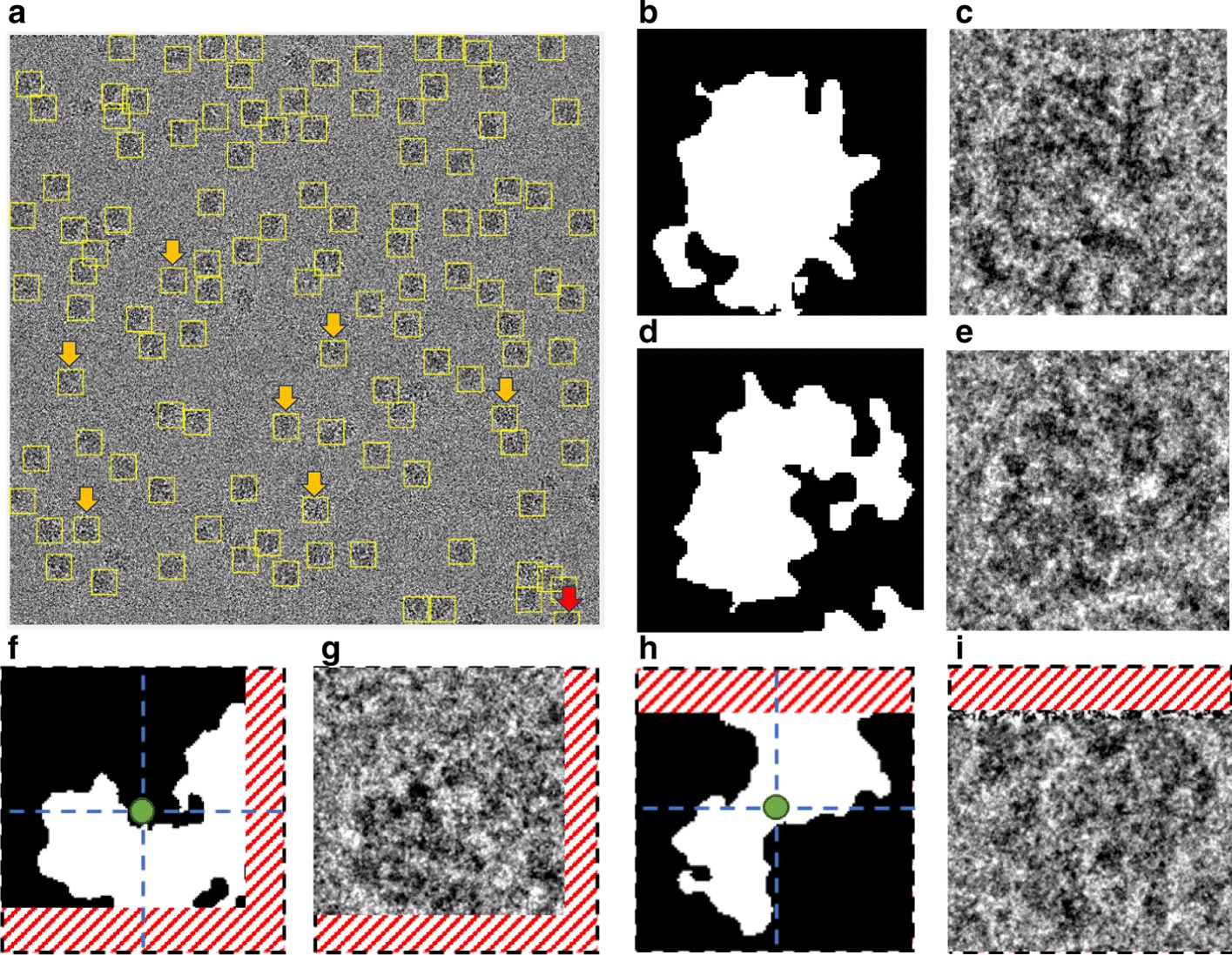
Fully automated irregular (complex) particles picking results using SuperCryoEMPicker approach [25] and good training particles-selection. **a** Particle detection and picking results using SuperCryoEMPicker approach [25] and cryo-EM micrographs form the Ribosome dataset [28]. **b**, **d** Good irregular particle binary mask examples. **c**, **e** Good training particle examples selection. **f**, **h** Bad irregular binary mask examples (dash red lines illustrate the missing part of the particle’s background while the dash blue lines illustrate the center of the object that the selected particle has to be in). **g**, **i** Bad particle examples (dash red lines illustrate the missing part of the particle’s background)

This step is also based on using the individual binary mask for each complex and irregular particle as shown in Fig. 17b, d, f, h. Then, we test each individual particle’s mask size and determine if it is a usable training sample. We develop a “good irregular (complex) training particle selection” algorithm (see Algorithm 2) to test each irregular binary particle, by calculating the average area for the whole particle binary masks which is determined by computing the total number of white pixels in each particle using the connected component particle mask’s pixel index list. Then the average area as is shown in Eq. (6):

6 $$Area = \frac{{\mathop \sum \nolimits_{l} allAreas}}{{\text{Total number of particles}}}$$

where $l$ is the total number of particles in each cryo-EM image.
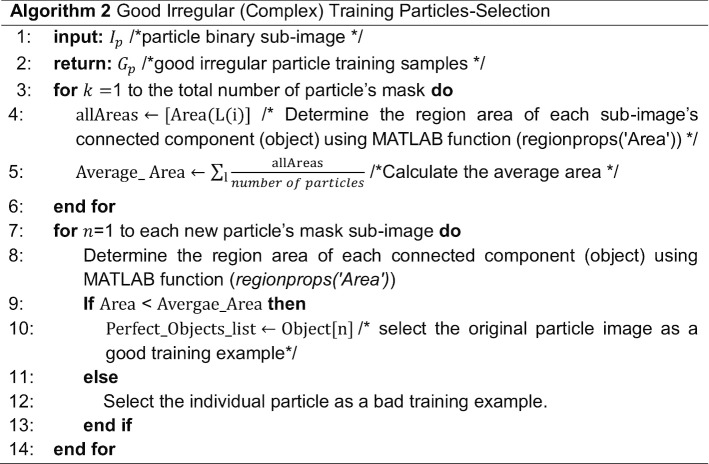

### Component 2: fully automated single particle picking based on deep classification network

**Fig. 18:**
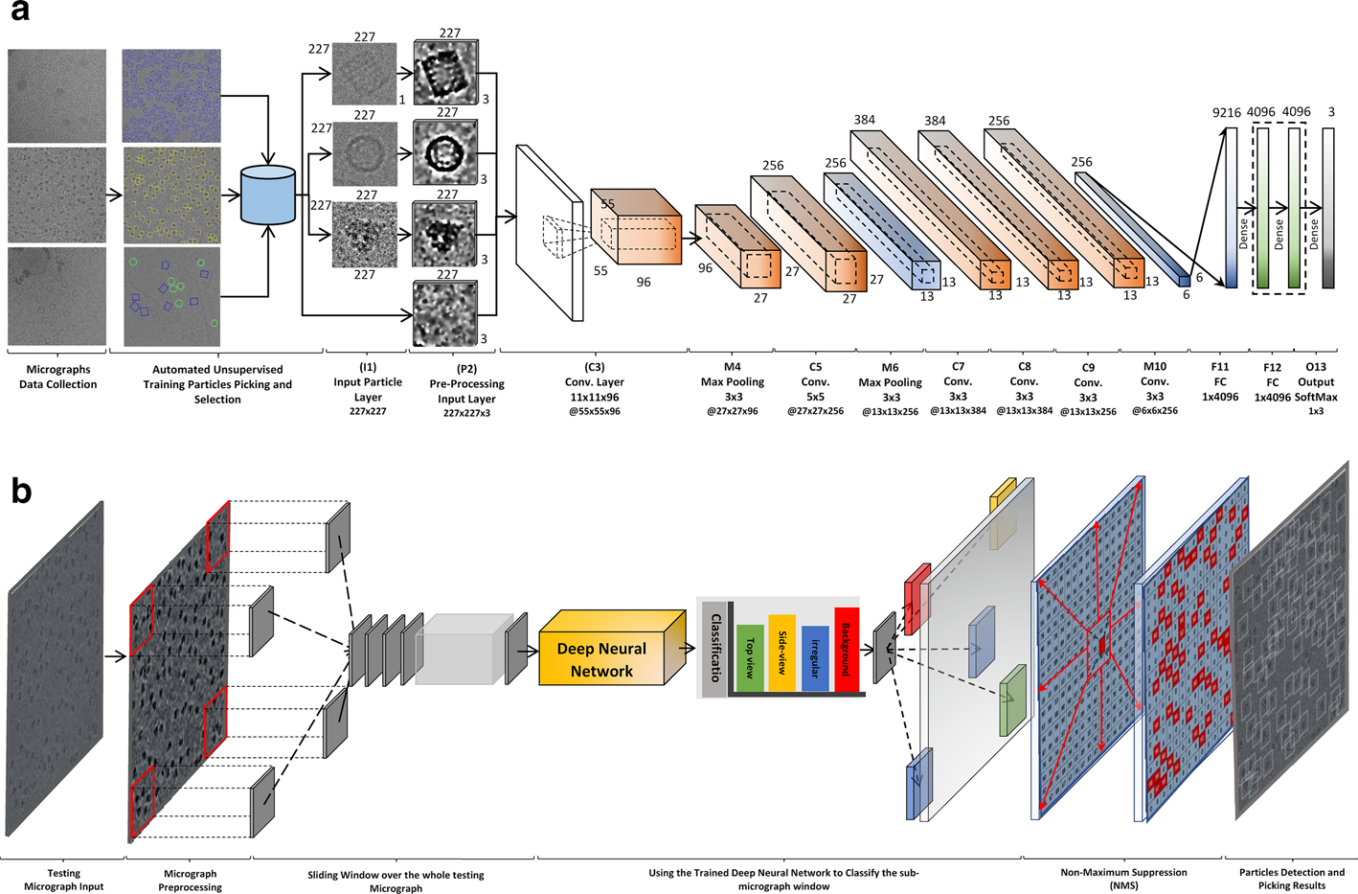
The architecture of the deep neural network used in DeepCryoPicker. **a** Training pipeline. The convolutional layer and the subsampling layer are abbreviated as C and S, respectively. C3:11 × 11 × 96 means that in the third convolutional layer (C3) is comprised of 96 feature maps, each of which has a size of 11 × 11, also. C3: @27 × 27 means that output feature maps dimensions are 27 × 27 pixels. **b** Testing pipeline

The second component of the DeepCryoPicker is the particle picking based deep neural network shown in Fig. 18. It consists of many layers such as the input layer, pre-processing layer, convolutional layers, sub-sampling layers, two fully connected layers, and one output layer. The main architecture of the DeepCryoPicker has in total thirteen layers as is summarized in Table 9. The first and second layers (input and the pre-processing layer) come from the first component of the DeepCryoPicker. The input layer takes the particles that have been already picked through the first model of the DeepCryoPicker. Each particle has been picked based on the preprocessed version of each of the micrographs. The rest are five convolutional layers, three max-pooling (subsampling) layers, two fully connected layers, and one output layer. To use one deep network structure, we unify the variety of the particle sizes as shown in Table 1 to one fixed size. In this case, after each particle is detected, a bounding box is drawn around each particle object in the cryo-EM image which is used to crop the particle image from the original micrograph. We recalculate the bounding box dimension of each detected particle after calculating the center of each box and specifying the fixed size of each (width and height). Then, the input size of the first and second layers (input and the preprocessing) in our DeepCryoPicker structure is $227 \times 227$. The third layer is the convolutional layer using 96 kernels with size $11 \times 11$. the first convolutional layer (third layer in the structure) produces 96 feature maps with dimensions $55 \times 55$. The fourth layer is the max-pooling layer with kernel size $3 \times 3$ and the feature maps output dimension is $27 \times 27$. The fifth layer is another convolutional layer using 256 kernels with size $5 \times 5$. The fifth layer (convolutional) produces 256 feature maps with dimensions $27 \times 27$. The sixth layer is another max-pooling layer with kernel size $3 \times 3$ and the feature maps dimensions output is $13 \times 13$. The seventh, eighth, and ninth layers are convolutional layers using different numbers of kernels 384, 384, and 256 respectfully. We use the same kernel size $3 \times 3$ for three convolutional layers. The output feature maps size for the last three convolutional layers $13 \times 13$. The tenth layer is the third max-pooling later with kernel size $3 \times 3$ and output dimensions $6 \times 6$. The last two layers are the fully connected layers to the final output (prediction layer) where the particle class is predicted based on the weight’s matrix and the activation function.

**Table 9:** The DeepCryoPicker network architecture

Layer	Type	Filters	Size
I1	Input layer	–	227 × 227 × 3
P2	Pre-processing	–	227 × 227 × 3
C3	Convolution	96	11 × 11
M4	Max-pooling	–	3 × 3
C5	Convolution	256	5 × 5
M6	Max-pooling	–	3 × 3
C7	Convolution	384	3 × 3
C8	Convolution	384	3 × 3
C9	Convolution	256	3 × 3
M10	Max-pooling	–	3 × 3
F11	Fully connected	–	1 × 4096
F12	Fully connected	–	1 × 4096
O13	Output	–	1 × 1

The convolutional layer and the subsampling layer are abbreviated as C and S, respectively. C3:11 × 11 × 96 means that in the third convolutional layer (C3) is comprised of 96 feature maps, each of which has a size of 11 × 11, also. C3: @27 × 27 means that output feature maps dimensions are 27 × 27 pixels

The convolutional and sub-sampling layers, which are core building blocks of the convolutional neural networks (CNN), produce feature maps. The kernel sizes are selected to establish the local connections while expanding through the entire particle image. The learnable kernels are convolved with each feature map from the previous layer. The convolutional layers (in the same convolutional operations) share the same local connective weights $W_{ij}^{\left[ l \right]}$ based on the previous layer’s weights $W_{ij}^{{\left[ {l - 1} \right]}}$, in which the feature maps in the current layer $X_{j}^{\left[ l \right]} { }$ are produced based on Eq. (7) [33]:

7 $$X_{j}^{\left[ l \right]} = {\text{s}}igmoid\left( {\mathop \sum \limits_{{i \in M_{j} }} X_{i}^{[l - 1} W_{ij}^{\left[ l \right]} + B^{\left[ l \right]} } \right)$$

where $l$ represents the convolutional layer, $W$ and $B$ is the shared weights and bias, $M$ is extracted feature maps (in the previous layer), $j$ is the output feature maps. Then, the feature maps are transformed to another layer by a non-linear activation function (sigmoid) as is given in Eq. (8) [6]:

8 $$Sigmoid\left( x \right) = \frac{1}{{1 + e^{ - x} }}$$

To reduce the positional over-fitting, the subsampling (max-pooling) layer is designed to subsample the input feature maps by decreasing the actual size and reduce the number of the parameters [33]: The max-pooling (subsampling) after each particular layer is computed based on Eq. (9) [33]:

9 $$X_{ij}^{\left[ l \right]} = \frac{1}{MN}\mathop \sum \limits_{m}^{M} \mathop \sum \limits_{n}^{N} X_{iM + m.jN + n}^{{\left[ {l - 1} \right]}}$$

where $I$ and $j$ are the positions of the output feature maps, $M$ and $N$ are the subsampling size. In the training process, the weights and bias are randomly initialized [0–1]. Then, they are updated during the training process. In our model, we used the cross-entropy loss function as the objective function Eq. (10) [34]:

10 $$L\left( w \right) = \sum\limits_{{i = 1}}^{N} {\sum\limits_{{c = 1}}^{C} { - y_{{ic}} logf_{c} \left( {x_{i} } \right) + \epsilon \left\| W \right\|_{2}^{2} } }$$

where $i$ is the sample number and c is its label, $x$ represents the predicted probability of the class $c$. $N$ is the total number of training samples, and $C$ is the total number of classes. During the training process, the errors of the objective function is minimized propagating error via the backpropagation algorithm based stochastic gradient descent as follow [35–37].

11 $$\omega \left( {l + 1} \right) = { }\omega \left( l \right) - \frac{\eta }{N}\mathop \sum \limits_{k = 1}^{N} {\mathcal{E}}_{n} \frac{{\partial {\mathcal{E}}_{n} }}{\partial }$$

where ${\mathcal{E}}$ is calculated as follow:

12 $${\mathcal{E}}_{n} = { }t_{n} - y_{n}$$

where $t_{n}$ is the label of the $n$th training sample, and $y_{n}$ is the value of the output layer corresponding to the $n$th training sample. $\omega \left( l \right)$ and $\omega \left( {l + 1} \right){ }$ represents the training parameter before and after the update of each iteration. The learning rate, $\eta$, is initially set to 0.0001.

## Data Availability

The datasets used in this study and the source code of DeepCryoEM are available at https://github.com/jianlin-cheng/DeepCryoEM.

## Abbreviations

Cryo-EM: Cryo-electron microscopy
AutoCryoPicker: An unsupervised learning approach for fully automated single particle picking in Cryo-EM images
SuperCryoPicker: A super-clustering approach for fully automated single particle picking in Cryo-EM
MRC: Medical research council
PNG: Portable network graphic
Micrograph: Digital image taken through a microscope

## References

[1] Han R, Wan X, Li L (2019). AuTom-dualx: a toolkit for fully automatic fiducial marker-based alignment of dual-axis tilt series with simultaneous reconstruction. Bioinformatics.

[2] Zhang Y, Sun B, Feng D, Hu H, Chu M, Qu Q, Tarrasch JT, Li S, Kobilka TS, Kobilka BK (2017). Cryo-EM structure of the activated GLP-1 receptor in complex with a G protein. Nature.

[3] Parmenter CD, Cane MC, Zhang R, Stoilova-McPhie S (2018). Cryo-electron microscopy of coagulation factor VIII bound to lipid nanotubes. Biochem Biophys Res Commun.

[4] Zhang J, Wang Z, Chen Y, Han R, Liu Z, Sun F, Zhang F (2019). PIXER: an automated particle-selection method based on segmentation using a deep neural network. BMC Bioinform.

[5] Frank J (2006). Three-dimensional electron microscopy of macromolecular assemblies.

[6] Zhu Y, Ouyang Q, Mao Y (2017). A deep convolutional neural network approach to single-particle recognition in cryo-electron microscopy. BMC Bioinform.

[7] Roseman AM (2003). Particle finding in electron micrographs using a fast-local correlation algorithm. Ultramicroscopy.

[8] Huang Z (2004). Application of template matching technique to particle detection in electron micrographs. J Struct Biol.

[9] Roseman AM (2004). FindEM- a fast, efficient program for automatic selection of particles from micrographs. J Struct Biol.

[10] Rath BK, Frank J (2004). Fast automatic particle picking from cryo-electron micrographs using a locally normalized cross-correlation function: a case study. J Struct Biol.

[11] Chen JZ, Grigorieff N (2007). SIGNATURE: a single-particle selection system for molecular electron microscopy. J Struct Biol.

[12] Langlois R (2014). Automated particle picking for low-contrast macromolecules in cryo-electron microscopy. J Struct Biol.

[13] Scheres S (2015). RELION: implementation of a Bayesian approach to cryo-EM structure determination. J Struct Biol.

[14] Adiga U (2005). Particle picking by segmentation: a comparative study with SPIDER-based manual particle picking. J Struct Biol.

[15] Woolford D (2007). SwarmPS: rapid, semi-automated single particle selection software. J Struct Biol.

[16] Yu Z (2004). Detecting circular and rectangular particles based on geometric feature detection in electron micrographs. J Struct Biol.

[17] Mallick SP (2004). Detecting particles in cryo-EM micrographs using learned features. J Struct Biol.

[18] Sorzano COS (2009). Automatic particle selection from electron micrographs using machine learning techniques. J Struct Biol.

[19] Tang G, Peng L, Baldwin PR, Mann DS, Jiang W, Rees I, Ludtke SJ (2007). EMAN2: an extensible image processing suite for electron microscopy. J Struct Biol.

[20] Wang F, Gong H, Liu G, Li M, Yan C, Xia T, Li X, Zeng J (2016). DeepPicker: a deep learning approach for fully automated particle picking in cryo-EM. J Struct Biol.

[21] Xiao Y, Yang G: A fast method for particle picking in cryo-electron micrographs based on fast R-CNN. In: AIP conference proceedings. AIP Publishing: 020080 (2017).

[22] Li H, Tian S, Li Y (2020). Modern deep learning in bioinformatics. J Mol Cell Biol.

[23] Li Y, Huang C, Ding L, Li Z, Pan Y, Gao X (2019). Deep learning in bioinformatics: introduction, application, and perspective in the big data era. Methods.

[24] Al-Azzawi A, Ouadou A, Tanner JJ (2019). AutoCryoPicker: an unsupervised learning approach for fully automated single particle picking in Cryo-EM images. BMC Bioinform.

[25] Al-Azzawi A, Ouadou A, Tanner JJ, Cheng J (2019). A super-clustering approach for fully automated single particle picking in cryo-em. Genes.

[26] N.d. KLH dataset. https://nramm.nysbc.org/.

[27] Grant T, Rohou A, Grigorieff N. EMPIAR-10146. 07 12; 2017. Accessed 03 Sept 2018.

[28] Wong W, Bai XC, Brown A, Fernandez IS, Hanssen E, Condron M, Tan YH, Baum J, Scheres SH (2014). Cryo-EM structure of the *Plasmodium falciparum* 80S ribosome bound to the anti-protozoan drug emetine. Elife.

[29] Scheres SH (2015). Β-galactosidase Falcon-II micrographs plus manually selected coordinates by Richard Henderson. J Struct Biol.

[30] Wang D, Li C, Wen S, Nepa S, Xiang Y. Daedalus: breaking non-maximum suppression in object detection via adversarial examples; (2019). arXiv:1902.02067v1. 10.1109/TCYB.2020.3041481 33400667

[31] Scheres SH (2012). RELION: implementation of a Bayesian approach to cryo-EM structure determination. J Struct Biol.

[32] Steve on Image Processing and MATLAB. Feret properties—wrapping up. Concepts, algorithms & MATLAB. https://blogs.mathworks.com/steve/2018/04/17/feret-properties-wrapping-up/.

[33] Waibel A (1989). Phoneme recognition using time-delay neural network. IEEE Trans Acoust Speech Signal Process.

[34] https://ml-cheatsheet.readthedocs.io/en/latest/loss_functions.html.

[35] Andrew N et al. Feature extraction using convolution; 2015. https://ufldl.stanford.edu/tutorial/supervised/FeatureExtractionUsingConvolution/.

[36] Rumelhart DE (1986). Learning representations by back-propagating errors. Nature.

[37] Langlois R (2011). A clarification of the terms used in comparing semi-automated particle selection algorithms in Cryo-EM. J Struct Biol.

[38] Koning R, Gomez-Blanco J, Akopjana I, Vargas J, Kazaks A, Tars K, Carazo J, Koster A (2016). Asymmetric cryo-EM reconstruction of phage MS2 reveals genome structure in situ. Nat Commun..

[39] Herzik MA, Wu M, Lander GCT (2017). Acidophilum 20S proteasome core movies obtained using Talos Arctica operating at 200 kV equipped with a K2 – image shift used for exposure target navigation. Nat Methods.

[40] Bartesaghi A, Merk A, Banerjee S, Matthies D, Wu X, Milne JL, Subramaniam S (2015). A resolution cryo-EM structure of β-galactosidase in complex with a cell-permeant inhibitor. Science.

